# Guidelines for Reducing the Adverse Effects of Shift Work on Nursing Staff: A Systematic Review

**DOI:** 10.3390/healthcare13172148

**Published:** 2025-08-28

**Authors:** Alessio Danilo Inchingolo, Angelo Michele Inchingolo, Maria Celeste Fatone, Laura Ferrante, Lucia Casamassima, Irma Trilli, Francesco Inchingolo, Andrea Palermo, Grazia Marinelli, Gianna Dipalma

**Affiliations:** 1 Department of Interdisciplinary Medicine, University of Bari “Aldo Moro”, 70124 Bari, Italy; alessiodanilo.inchingolo@uniba.it (A.D.I.); angelomichele.inchingolo@uniba.it (A.M.I.); laura.ferrante@uniba.it (L.F.); lucia.casamassima@uniba.it (L.C.); irma.trilli@uniba.it (I.T.); graziamarinelli@live.it (G.M.); gianna.diplama@uniba.it (G.D.); 2 Department of Biomedical, Surgical and Dental Sciences, Milan University, 20122 Milan, Italy; 3 PTA Trani-ASL BT, Viale Padre Pio, 76125 Trani, Italy; maria.fatone@aslbat.it; 4 Department of Experimental Medicine, University of Salento, 73100 Lecce, Italy; andrea.palermo@unisalento.it

**Keywords:** education, healthcare improvement, psychotherapy, mental health, nurses, occupational risks, rostering, shift work, sleep hygiene

## Abstract

Background: The increasing demand for care in hospital settings, often at a high intensity, requires organizing work according to 24 h shifts. Nevertheless, shift work (SW), especially at night, alters the circadian rhythm, negatively affecting the psychophysical health of nurses, compromising their quality of life, and jeopardizing patient safety. Shift-work-related diseases (SWDs) can arise from these disruptions. Methods: This systematic review aims to evaluate the effects of several types of medical, psychotherapeutic, and educational interventions and strategies on shift-work-related diseases (SWDs). The databases PubMed, Embase, Web of Science, and Cochrane were searched using the MESH terms “shift work” and “nurses” from January 2015 to March 2025. A total of 43 articles were included in the final analysis. Results: Quantitative findings from the studies showed, for example, improvements in sleep quality scores ranging from 15% to 40% with optimized shift planning, reductions in fatigue scores by 20–35% through strategic napping, and moderate effect sizes for light therapy interventions. Physical activity and relaxation techniques were associated with a 10–25% improvement in subjective well-being indices, while meal timing interventions led to reductions in gastrointestinal symptom prevalence by up to 18%. The selected articles were discussed by dividing them according to the type of intervention applied to shift nurses, namely improvement of shift planning, light and temperature modulation, introduction of napping, supplementation, meal management, psychotherapy, sleep education, physical activity, relaxation techniques and yoga, music therapy, and aromatherapy. This categorization was performed to highlight the range of strategies tested and their relative quantitative impact. Conclusions: There is evidence that SWDs can be mitigated through targeted interventions and strategies. The limitations of the studies examined include small sample sizes, extreme heterogeneity of follow-up, the few numbers of randomized controlled trials, and the prevalence of female or Intensive Care Unit nurses in study samples. Further research should focus on large-scale randomized controlled trials, multicenter longitudinal studies, and the evaluation of the most promising interventions—particularly light therapy, optimized shift scheduling, and structured napping protocols—to assess their long-term efficacy and generalizability.

## 1. Introduction

Shift work (SW) refers to any work schedule that falls outside the traditional daytime hours of 7 a.m. to 6 p.m. It includes evening, night, and rotating shifts, which are common in industries requiring 24 h operations, such as healthcare, law enforcement, transportation, and emergency services [1]. In nursing, SW is essential to ensure continuous patient care, but it often leads to disruptions in circadian rhythms, sleep deprivation, and fatigue [2]. These factors can negatively impact nurses’ health, increasing the risk of stress, metabolic disorders, cardiovascular diseases (CVDs), and reduced cognitive performance [3]. Additionally, SW affects social and family life, contributing to burnout and job dissatisfaction. To mitigate shift-work-related diseases (SWDs), proper scheduling strategies, sufficient rest periods, and ergonomic work arrangements are crucial in improving nurses’ well-being and maintaining high-quality patient care [4].

SW has significant effects on sleep, mainly due to disruptions in the natural circadian rhythm, leading to various health issues, including sleep disorders like insomnia and excessive daytime sleepiness [5]. Nursing staff working in a two-shift system often experience these disturbances due to the demands of night shifts, which suppress melatonin production, a hormone crucial for regulating sleep. The most notable SWDs are often related to sleep quality, with individuals reporting difficulty both falling asleep and maintaining sleep, particularly when transitioning between day and night shifts. This leads to sleep deprivation, which is linked to an increased risk of CVDs, metabolic issues, and even certain types of cancers, such as breast cancer in women, as SW has been recognized as a potential carcinogen by the International Agency for Research on Cancer. Moreover, the sleep disturbances caused by SW can have a detrimental impact on job performance, increasing fatigue and lowering cognitive function, which could affect the quality of healthcare services provided [6,7,8,9,10].

The effects of SW on the cardiovascular system have been widely studied because long-term exposure to night SW increases the risk of hypertension and coronary heart disease (CHD) [11]. Specifically, working more than five night shifts per month and slowly rotating shifts (that is, always attending the same shift for a certain period of time rather than changing shifts quickly in a week) significantly increased the likelihood of hypertension [12]. A prospective cohort study of female nurses showed that the duration of rotating night SW was positively correlated with an elevated risk of CHD. This association was more pronounced in the early years of exposure and persisted even after adjusting for other risk factors [13,14,15,16]. In addition, the adverse effects of SW on the cardiovascular system accumulate over time, with a significant increase in CHD risk observed in those who worked rotating night shifts for extended periods [17,18,19,20,21].

It has been found that sleep disturbances, poor sleep quality, and sleep deprivation negatively affect metabolic health, increasing the risks for obesity, type 2 diabetes, hypertension, and metabolic syndrome (MetS) [22,23,24,25,26].

Moreover, nurses working shift schedules tend to develop irregular eating habits, poor dietary choices, and lower physical activity levels, which further exacerbate metabolic issues such as weight gain and higher body fat percentages. The relationship between SW and metabolic dysfunction is complex and multifactorial, involving both direct physiological effects, such as hormonal imbalances (e.g., altered cortisol and insulin levels), and indirect lifestyle factors, such as disrupted eating patterns and physical inactivity. Irregular sleep patterns and inconsistent meal timings are significant contributors to these metabolic issues [27,28,29]. Nurses who consume a higher proportion of their daily caloric intake after 7 p.m., often due to irregular work hours, tend to have a higher prevalence of MetS [26,30,31,32,33].

Night SW has been associated with an increased risk of various cancers, including breast and colorectal cancer [34]. Long-term exposure to circadian disruption can affect processes such as cell proliferation and DNA repair, as well as the suppression of melatonin, a hormone with potential anticancer properties [35,36,37,38,39]. While evidence for a link between night SW and breast cancer is relatively stronger, studies on other cancers like colorectal and thyroid cancer remain inconclusive, with some suggesting a modest increase in risk associated with sleep difficulties among night shift workers [40]. However, the overall evidence for a direct causal relationship between SW and cancer risk remains weak, and further research is needed to better understand the mechanisms involved [41,42,43,44,45].

Night SW increases the risk of several neurological diseases, altering sleep circadian rhythms and melatonin levels, which are crucial for cognitive health. Disruption of these processes has been associated with cognitive decline, including impairments in working memory and attention, with night shifts causing the most significant decline in these areas [46]. Additionally, prolonged exposure to night SW, particularly lasting 20 or more years, has been linked to an increased risk of multiple sclerosis (MS) [47,48]. While the overall evidence does not consistently support a direct link between SW and MS, long-term exposure may contribute to the onset of definite MS [49].

SW significantly affects psychosocial well-being in nursing, with nurses working irregular or rotating shifts experiencing higher levels of psychological diseases, such as anxiety, depression, and stress [50,51,52,53,54,55,56]. These conditions are worsened by long working hours and disrupted sleep patterns, contributing to burnout and fatigue. Persistent night SW is linked to increased mood and neurotic disorders [57]. Additionally, factors like low social support, lack of control over shift schedules, and inadequate staffing worsen these psychosocial risks [58]. SW also impacts nurses’ work-family balance, with rotating shifts leading to more work-family conflicts and reduced work–life quality [59]. Nurses with more control over their shift schedules report lower burnout and exhaustion [60,61,62,63,64,65]. Hence, SW poses significant psychosocial risks for nurses, affecting their mental health and job performance [66,67,68].

**Figure 1:**
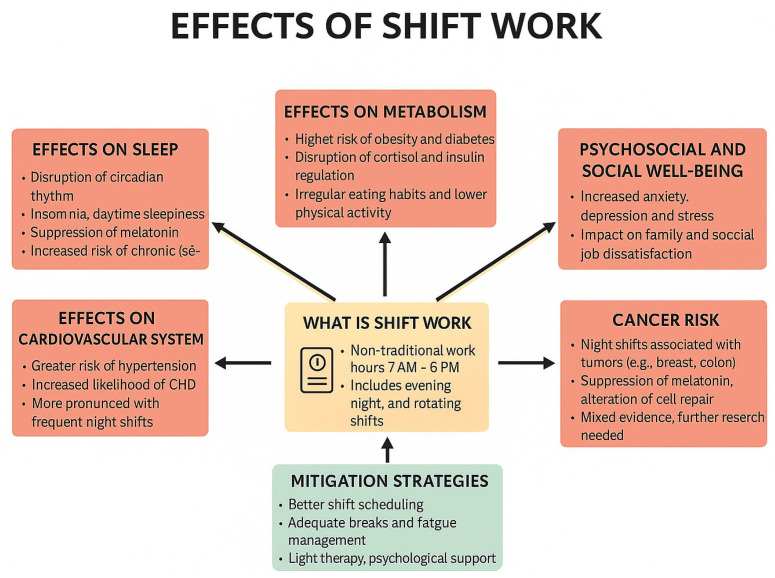
A visual guide of how shift work (SW) affects health and well-being. The red boxes illustrate the adverse health and psychosocial effects associated with shift work, whereas the green box presents evidence-based mitigation strategies aimed at reducing these negative outcomes.

On the other hand, SW has significant effects on patient safety, primarily due to the impact on healthcare workers’ fatigue, sleep deprivation, and overall alertness. Particularly, SW extended 12 h or longer contributes to physical and mental exhaustion, which increases the likelihood of medical errors, including medication mistakes and procedural lapses. Studies show that fatigue leads to slower reaction times, decreased attention, and a higher risk of oversight in patient care [67,69,70,71,72]. Additionally, sleep disruption, particularly from night SW, can impair cognitive function and increase the chance of errors. The cumulative effect of long shifts and irregular working hours compromises the quality of care, as healthcare workers are less able to stay focused and make critical decisions. Herein, while SW is essential for providing 24/7 healthcare, its negative impact on worker well-being poses a significant risk to patient safety, giving rise to the need for better management of shift patterns and work hours in healthcare settings (Figure 1) [68,73,74,75,76].

This systematic review aims to evaluate the effectiveness of medical, psychotherapeutic, and educational approaches to mitigate nurses’ SWDs. In addition, evidence-based guidelines were provided to help healthcare institutions implement more effective measures to support shift-working nurses, ensuring their well-being and improving patient care quality.

## 2. Materials and Methods

### 2.1. PICO Question

“Can shift-working nurses (P) benefit from various strategies and interventions (I) compared with the control group (C) in terms of improvement of health and quality of life and occupational risk reduction (O)?” (Population: nurses on SW; Intervention: medical, psychotherapeutic, and educational interventions and strategies to reduce SWDs and improve quality of life; Comparison: control group of nurses on SW who do not implement such strategies and interventions (may be absent in some articles); Outcome: improved health and quality of life for shift nurses, reduced occupational risks.)

### 2.2. Research Protocol

This systematic review was conducted following PRISMA (Preferred Reporting Items for Systematic Reviews and Meta-Analysis) 2020 guidelines and registered in the International Prospective Register of Systematic Review Registry guidelines (PROSPERO; ID: 1014195; https://doi.org/10.1136/bmj.n71).

### 2.3. Search Processing

Four electronic databases, namely PubMed, Scopus, Web of Science, and Cochrane, were consulted to find articles that match our topic, dating from 1 January 2015 to 3 March 3 2025. The following Medical Subject Heading (MESH) terms were used: “shift work” AND “nurses” (Table 1).

### 2.4. Inclusion and Exclusion Criteria

Articles were selected if they met the following inclusion criteria: (1) human subjects; (2) English language; (3) randomized controlled trials, clinical trials, cohort studies, and observational studies; (4) nurses performing SW; (5) research time limits; and (6) open access.

The exclusion criteria applied were the following: (1) other languages except English; (2) reviews, meta-analyses, and case reports; (3) population not relevant; and (4) outcomes not relevant.

### 2.5. Data Processing

Three reviewers (L.C., L.F., and I.T.) independently screened the records according to the inclusion criteria. The variables extracted were the characteristics of the study population (both male and female shift nurses of all ages), the interventions to mitigate nurses’ SWDs (type, dosage, duration, frequency), the control group (shift work nurses who do not receive such interventions), methodological aspects (study design, sample size, analysis method, risk of bias), and outcomes (type of SWD improved, measurement of effects on study population, duration of follow-up). The reviewers crosschecked each other’s selected articles. The discrepancies were resolved through root cause analysis, data cleaning and transformation, re-extraction of data in some cases, documentation of the discrepancies to ensure transparency and future reference, and an expert consultation (F.I.).

The selected articles were downloaded into Zotero (version 6.0.15).

### 2.6. Quality Assessment

The quality of the included papers was assessed by two reviewers, M.C.F. and G.D., using the ROBINS-I, which is a tool developed to assess the risk of bias in the results of non-randomized studies that compare the health effects of two or more interventions. Seven domains were evaluated, and each was assigned a degree of bias, namely (1) bias due to confounding, (2) bias in selection of participants into the study, (3) bias in classification of the intervention, (4) bias due to deviations from intended interventions, (5) bias due to missing data, (6) bias in measurement of the outcome, and (7) bias in selection of the reported result. A third reviewer (F.I.) was questioned in case of any doubt.

## 3. Results

### 3.1. Characteristics of the Included Articles

A total of 460 records were identified using the keywords “shift work” and “nurses.” When applicable, the automatic filters entered were as follows: human subjects, only English articles, only clinical studies, and no reviews. The databases consulted were PubMed (139), Scopus (99), Web of Science (185), and Cochrane (37). All records were uploaded to an Excel file.

During the screening phase, inclusion and exclusion criteria were applied based on title and abstract analysis. Only studies focusing on strategies and interventions that can be implemented to reduce the effects of SW on nurses were selected. Studies concerning the effects of SW on nurses but focusing on other areas, such as negative effects on nurses’ mental and physical health, increased risks to the patient, and advantages and disadvantages of SW on nurses’ organization of daily life, were considered off-topic. After screening, 347 articles were excluded by analyzing the title and abstract, leading to 113 records. Then, duplicates (36) were removed manually, resulting in the selection of 77 records. Among the included studies, both interventional and observational designs (longitudinal and cross-sectional) were considered eligible if they evaluated strategies or interventions targeting the effects of shift work.

**Figure 2:**
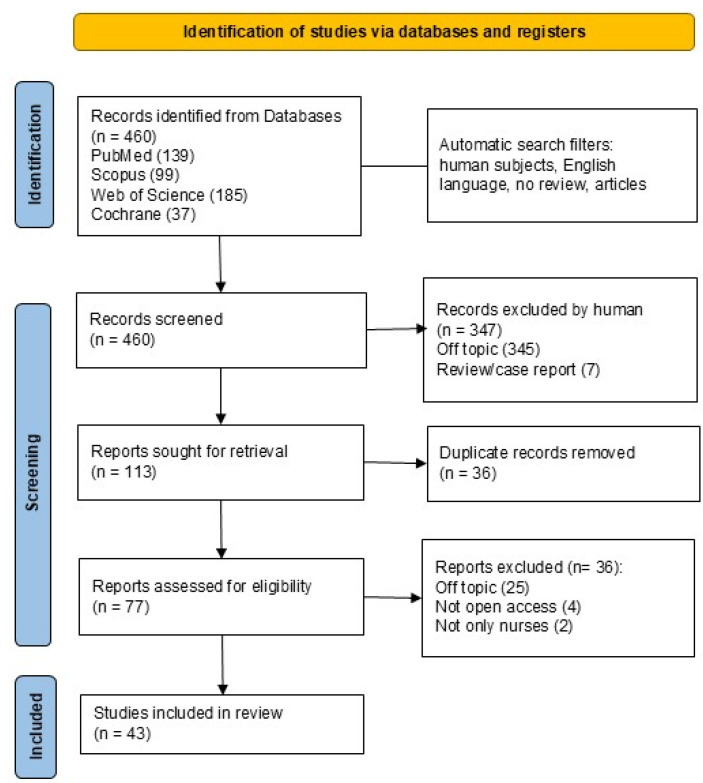
PRISMA (Preferred Reporting Items for Systematic Reviews and Meta-Analysis) 2020 flowchart (https://doi.org/10.1136/bmj.n71).

At the end of the eligibility process, 43 studies were included in the final analysis. The process is illustrated in the flowchart of Figure 2, and in Table 2 there is a summary of Interventions and Main Outcomes.

### 3.2. Quality Assessment and Risk of Bias of Included Articles

The risk of bias assessment, in Table 3, conducted with the ROBINS-I tool on the 43 included studies showed that most presented a moderate risk in at least two domains, particularly bias due to confounding and bias in measurement of outcomes, where over 60% of studies raised concerns. This finding reflects the frequent lack of adequate control for potential confounding variables and the predominant use of subjective assessment tools (questionnaires and self-reports) rather than objective measures. Bias in the classification of interventions and bias due to deviations from intended interventions were the domains with the highest proportion of high risk, especially in studies without clearly defined intervention protocols. Missing data was a relevant issue in about one-third of the studies, with potential implications for internal validity and generalizability. Only the studies by Pahlevanzadeh M.J. et al. and de Bruijn L. et al. showed a low risk of bias across all domains, indicating strong methodological rigor. At the opposite extreme, seven studies reported high risk in multiple domains, often associated with deviations from intended interventions and inaccurate intervention classification. Overall, these results suggest that the conclusions of this review should be interpreted with caution: although positive evidence was identified for several strategies, the overall strength of the evidence is limited by the heterogeneous methodological quality of the available studies.

The following tables show the characteristics of the selected studies and their relevant findings, divided by SWD improvement intervention, namely (1) the effects of work schedules, flexibility, and recovery time; (2) the effects of individual tolerance factors and chronotype; (3) the effects of satisfaction questionnaires; (4) the effects of light and temperature modulation; (5) the effects of supplementation; (6) the effects of meal management; (7) the effects of physical activity, relaxation techniques, and yoga; (8) the effects of physiotherapy; (9) the effects of sleep education; and (10) the effects of music therapy and aromatherapy.(Table 4, Table 5, Table 6, Table 7, Table 8, Table 9, Table 10, Table 11, Table 12 and Table 13).

## 4. Discussion

### 4.1. Shift Planning

#### 4.1.1. Work Schedules, Flexibility, and Recovery Time

Several studies have investigated the planning of shift schedules and how different models of nurse rostering impact nurse performance, engagement, and fatigue. Many authors agree that excessively long shifts should be avoided, and an adequate number of days off should be allowed after a night shift. Dall’Ora et al. reported in their cross-sectional study that 12 h shifts reduced opportunities for continuing education, patient discussions, and continuity of care, highlighting a trade-off between staffing efficiency and quality of care [79]. Niu et al., in the prospective longitudinal, parallel-group comparative study, reinforced the biological difficulty of adjusting to night shifts, demonstrating that cortisol rhythms require at least two days off to normalize [77]. These findings support the importance of allowing sufficient recovery time and structuring shifts around the natural human circadian rhythm [77]. Complementing these findings, Kubo et al., in a non-randomized controlled cross-over study, tested a targeted intervention by extending restart breaks after consecutive night shifts [82]. Allowing extended restart breaks from 31 to 55 h after consecutive night shifts led to reduced subjective fatigue and psychological distress, although no significant improvements were observed in objective physiological markers, namely vital exhaustion, distress, hair cortisol, salivary C-reactive protein, and sleep mattress sensor sensation [82]. Likewise, Waage et al. demonstrated in a longitudinal cohort study that reducing night shifts in the last year or eliminating quick returns significantly improved outcomes for nurses suffering from SWDs, thereby reinforcing the importance of tailoring schedules to individual biological predispositions [81]. In comparison, Shiffer et al. pointed out in a cross-sectional study the significance of rotation direction: clockwise schedules were associated with better sleep quality and work–life balance compared to counterclockwise rotations [78]. Conversely, Inoue et al. presented in a survey-based observational study a more optimistic perspective: under variable shift systems—particularly those structured with restorative breaks and opportunities for social interaction—nurses reported sustained work engagement [78]. According to the authors, the variable shift system proves effective, and nurses should be allowed to opt for a break based on conversation or rest [80].

#### 4.1.2. Individual Factors and Chronotype in Shift Tolerance

Nursing managers should consider individual nurse characteristics and their preferences to be able to arrange tailor-made shifts. For this purpose, they can use the help of mathematical models, digital media, and surveys. Self-scheduling of shifts should also be encouraged. A key characteristic to consider is the chronotype. Chronotype, genetically defined and modulated by the environment, correlates with individual preferences for sleep–wake rhythms and depends on the secretion of cortisol and melatonin from the hypothalamic-pituitary axis. There are three main categories of chronotype: morning types, evening types, and neither type, the latter being the most adaptable to various shifts.

In two descriptive cohort studies, Bülbül et al. and de Bruijn et al. both found that evening-type nurses, despite being more likely to work night shifts, experienced poorer sleep quality and lower overall well-being. In contrast, intermediate chronotypes performed better, as their natural rhythms aligned more closely with typical shift patterns [85,87]. Therefore, chronotype is associated with shift type preference and sleep disorders in nurses [85,87]. These findings are consistent with the results of the cross-sectional study by Jung et al., who emphasized that SW tolerance is influenced by a combination of individual tolerance factors, namely age and number of children, chronotype, job stress, social support, and lifestyle habits, indicating that adaptation is multifactorial rather than only schedule dependent [85,87]. Individual SW tolerance factors can influence insomnia, depression, and fatigue. Similarly, in a multicenter cross-sectional study, Li et al. found that work-related quality of life and chronotype were stronger predictors of sleep quality than the specific shift pattern itself among maternal and child health nurses [86]. Nursing managers should take into account nurses’ chronotype and quality of work life and make interventions that affect both sleep-related modifiable factors (such as frequent caffeine intake and irregular meals) and non-modifiable factors (e.g., age and chronotype) [86].

Pahlevanzadeh et al. proposed a justice-based scheduling model that incorporated nurse preferences and performance metrics, based on a mathematical model of integer binary programming [84]. After the application of this mathematical model, the tax of absenteeism and the number of performance complaints decreased by 40% and 50%, respectively. In parallel, nurses’ satisfaction increased by 30%. This suggests that, in some cases, personal and psychosocial factors may have a greater impact than structural scheduling characteristics [84].

In conclusion, these studies underscore the potential benefits of chronotype-aware scheduling and tailor-made interventions as strategies for improving nurses’ health, sleep quality, and overall work performance.

#### 4.1.3. Satisfaction Questionnaires

The role of the organizational environment has emerged as a critical factor influencing SWDs. According to the cross-sectional study by Dehring et al., organizational climate significantly impacts nurse health; notably, rotating shift workers reported the highest levels of psychological distress [88]. However, supportive factors such as supervisor engagement and task orientation were found to buffer these negative effects [88]. Similarly, Abed Al Ahad et al., in a longitudinal study, investigated shift-specific job satisfaction and found that satisfaction levels were highest when nurses experienced manageable workloads and minimal rationing of care [89]. Together, these studies highlight the importance of organizational support, continuous workload monitoring, and evidence-informed scheduling practices in promoting a healthier and more sustainable SW environment [89]. Consistent with these concerns, Lee et al. reported in a descriptive study on compliance with ergonomic scheduling guidelines in South Korea, primarily due to institutional barriers and a lack of formal training for staff involved in roster creation [90]. In addition, in a qualitative single case study, Booker et al. also revealed widespread under-preparation among scheduling personnel, where fatigue-related risks were frequently neglected in favor of lifestyle-oriented self-rostering [91]. While self-rostering may offer greater flexibility, it can inadvertently lead to increased fatigue if not accompanied by proper guidance and fatigue risk management strategies grounded in scientific evidence [91]. The methodological study for instrument development by Shin et al. focuses on developing and validating a tool to assess the quality of a healthy work environment for shift nurses in South Korea [92]. Based on frameworks from the World Health Organization and the American Association of Critical-Care Nurses, the authors created a 23-item instrument covering five key areas: physical gratification, psychological stability, independent competency, collaborative relationships, and structural support [92]. The tool was found to be reliable and valid, showing good internal consistency and test–retest reliability. Its goal is to help improve nurse well-being, reduce burnout, and ultimately enhance patient care by identifying and improving aspects of the work environment [92].

### 4.2. Light and Temperature Modulation

It has been shown that intense light, even of short duration, confers acute positive effects on nurses working night shifts, including increased subjective alertness and a reduction in insomnia, anxiety, and depression [117]. Griepentrog et al. conducted a randomized crossover trial exposing Intensive Care Unit (ICU) nurses to 1500–2000 lux white light during 10 h of the night shift [93]. They found a significant reduction in subjective sleepiness but a paradoxical increase in psychomotor errors, suggesting that while alertness improved, cognitive precision may have declined under intense lighting [93]. In contrast, Bjorvatn et al. examined the effects of timed bright light exposure (10,000 lux) during three consecutive night shifts. Their placebo-controlled crossover study found that bright light reduced “heavy eyelids” (a subjective marker of sleepiness) but did not improve performance on the Karolinska Sleepiness Scale (KSS) or the Psychomotor Vigilance Task [94]. Moreover, no significant changes were observed in post-shift recovery, highlighting the limited long-term benefit of bright light when poorly timed or inconsistently applied. The study by Bjorvatn et al. (2021) emphasizes that bright light exposure (10,000 lux) can help delay the circadian rhythm, especially when administered progressively later across consecutive night shifts—from 2:00 to 3:00 on the first night, 3:00–4:00 on the second, and 4:00–5:00 on the third [94]. While both studies report short-term alertness benefits, they differ in terms of how well those benefits translate into functional outcomes such as vigilance and error rates.

Opposing the bright light paradigm, Hoshi et al. explored whether minimum night lighting (110 lux) could better support circadian alignment [95]. Their quasi-experimental study found that fatigue and sleepiness were significantly higher under dim lighting compared to brighter conditions (410 lux), with no improvement in post-shift sleep quality [95]. Importantly, no significant changes were found in patient safety incidents, suggesting that dimmer lighting may reduce visual alertness without offering meaningful physiological recovery. The study by Hoshi et al. found that working under dim light conditions (<120 lux) during the night shift did not improve fatigue, sleepiness, or sleep quality compared to brighter conditions (~410 lux). Importantly, light levels above 120 lux—even for short durations—were shown to suppress melatonin and disrupt circadian rhythms. [95]. This contrasts with the intended physiological rationale: that reduced lux levels might help maintain melatonin rhythms and improve adaptation. However, the lack of measurable benefits in fatigue or sleep, combined with the practical challenges of navigating darker environments, suggests that minimum lighting strategies may be less viable for safety-critical environments like hospitals.

Besides light, the other environmental factor that can be easily changed in work environments is temperature. It was discovered that preset hospital temperatures vary between 20 °C and 25 °C and temperatures above 25 °C can cause discomfort, impairing work performance in hospital settings. Furthermore, it has been found that the body temperature is lower at night due to circadian rhythms [92]. Kim et al. explored the effects of ambient temperature (23 °C vs. 26 °C) on night-shift nurses [96]. Their crossover study found that thermal comfort improved significantly at 23 °C, as did body temperature regulation, though there were no significant differences in fatigue, sleepiness, or adaptation scores between temperature conditions. Interestingly, urinary melatonin levels dropped more sharply at 23 °C, indicating a possible trade-off between physical comfort and hormonal circadian rhythms [96].

### 4.3. Supplementation

The supplements evaluated for mitigating sleep disturbances in shift-working nurses are melatonin and zinc [97,98]. In an RCT, Sadeghniiat-Haghighi et al. evaluated the effects of 3 mg of melatonin in shift workers with delayed sleep onset. Melatonin supplementation was administered 30 min before the workers’ usual sleep time following their night shift. The intervention significantly reduced sleep onset latency and improved sleep efficiency, though it had no measurable effect on total sleep time or wakefulness after sleep onset [97]. These results support melatonin’s short-term utility in addressing circadian misalignment, particularly in individuals with symptom-specific sleep initiation issues. However, the study’s generalizability is limited by the absence of long-term follow-up and its reliance on actigraphy rather than more robust objective sleep measures like polysomnography [97].

Gholipour Baradari et al. examined the role of zinc supplementation (220 mg every 72 h for one month) in a sample of ICU nurses with both poor sleep quality and low serum zinc levels. The study reported significant improvements in subjective sleep quality and sleep latency, alongside a notable increase in serum zinc concentration [98]. These findings suggest a biological link between micronutrient status and sleep regulation, consistent with prior research on zinc’s antioxidant and neuromodulatory roles. Unlike the melatonin study, which targeted circadian regulation, this intervention addressed nutritional deficiency as a potential root cause of sleep disturbance. Zinc supplementation was administered shortly before the sleep episode, typically between 9:00 p.m. and 11:00 p.m. [98].

Critically, while these interventions yielded positive short-term results, they differ in mechanism and application: melatonin primarily affects circadian rhythm realignment, while zinc appears to influence sleep quality through physiological restoration and nutritional correction.

### 4.4. Meal Management

Nutrition during night shifts is a crucial factor for the well-being and performance of healthcare workers, as it directly affects circadian rhythm regulation, blood glucose levels, and overall physical and psychological health.

The recent RCT by Suyoto et al. examined the impact of glycemic index (GI) and meal frequency on glycemic control in a sample of night-shift nurses. The findings highlighted that the quality of carbohydrates consumed plays a more critical role than meal frequency. Specifically, consuming either one or three high-GI meals during the night led to a significant increase in blood glucose levels and glycemic variability, whereas low-GI meals—even when eaten more than once and without fasting—did not negatively affect the glycemic profile. These results suggest that, during night shifts, favoring low-GI foods can help prevent metabolic disturbances, avoiding both prolonged fasting and the glycemic spikes associated with simple sugars [99].

In parallel, the RCT by Leedo et al. assessed the impact of providing healthy meals at the workplace on mood, reaction time, and dietary intake among both daytime and shift healthcare workers. Although no significant changes were found in reaction time, shift workers reported notable improvements in mood during the intervention period, including reduced fatigue, increased vitality, and an overall improvement in emotional well-being. Additionally, the intervention led to healthier eating habits, with increased intake of fiber, complex carbohydrates, and water, and a reduction in fat consumption [100]. A study by Molzof et al. found that higher food intake during night shifts was associated with increased lipid levels, regardless of macronutrient composition, highlighting the role of meal timing in cardiometabolic risk among shift-working nurses [116].

Selecting low-GI foods can help stabilize blood glucose levels and prevent fluctuations that impair alertness and performance. In addition, the availability of balanced and hydrating meals in the workplace can positively influence mood, an especially relevant factor in emotionally demanding clinical environments.

### 4.5. Physical Activity, Relaxation Techniques, and Yoga

Structured physical activity has shown promising results in reducing stress, preventing musculoskeletal disorders, and enhancing overall quality of life. Several studies have investigated different modalities of exercise—supervised, home-based, or mind–body oriented—adapting them to the specific demands of SW.

The RCT by Matsugaki et al. highlighted the benefits of a workplace exercise program supervised by physical therapists for shift-working nurses. After 12 weeks of aerobic and resistance training, the supervised group demonstrated significant improvements in aerobic capacity, muscular strength, and several biochemical markers (such as high-density lipoprotein cholesterol and adiponectin), along with a notable reduction in depressive symptoms. Although the adherence rate was similar between the supervised and voluntary (self-guided) groups, the superior outcomes in the supervised group suggest that the presence of a physiotherapist provided essential motivational and technical support, enhancing the effectiveness of the intervention [101].

The recent experimental study by Baek et al. evaluated a smartphone-based home workout program developed to promote physical activity during the Coronavirus Disease 2019 (COVID-19) pandemic. Participants reported significant improvements in sleep quality, fatigue, musculoskeletal symptoms, and psychological resilience, further confirming the value of digital health programs that combine accessibility with motivational support. While no significant changes were observed in work performance, the intervention demonstrated the potential of mobile-based solutions as sustainable and customizable tools to enhance nurse well-being, particularly during times of health system strain [102].

A more relaxation-focused approach was explored by the randomized crossover trial by Miyoshi, who investigated the effects of restorative yoga on SW nurses. After four weeks of regular home practice, participants reported a significant reduction in perceived stress compared to their usual stress management methods (such as sleeping, shopping, or socializing). Although no significant changes were observed in vital signs, the subjective improvement in well-being suggests that restorative yoga may positively impact psychological health, offering an accessible and autonomous strategy for coping with occupational stress [103].

Targeted physical activity interventions—despite their differences in delivery and structure (at home or during SW)—can significantly benefit the health of SW nurses. Professional supervision, mobile health technologies, and flexible mind–body practices emerge as key factors in facilitating their adoption, adherence, and sustainability.

### 4.6. Psychotherapy

In the last two years, several authors have explored innovative psychotherapy approaches—often combining digital technologies and cognitive strategies—to reduce burnout, improve sleep quality, and strengthen emotional regulation.

The RCT by Baek et al. tested the effectiveness of a personalized artificial intelligence (AI)-assisted intervention for reducing burnout among nurses. The algorithm tailored the intervention content—which included mindfulness, Acceptance and Commitment Therapy, reflective writing, and laughter therapy—based on each participant’s individual psychological profile. Results showed a significant reduction in burnout, especially in dimensions related to patient interaction, highlighting the potential of intelligent technologies as flexible and scalable tools to support the psychological health of healthcare workers [104].

Similarly, the RCT by Ell et al. investigated the efficacy of digital cognitive behavioral therapy for insomnia. The intervention led to significant improvements in sleep quality and sleep efficiency and a reduction in psychological distress. Participants also reported improved daytime functioning, suggesting that well-designed digital solutions can serve as practical resources for addressing sleep disorders among shift workers [105].

A more comprehensive approach was adopted by Wenhua Liu et al. through a Complex Interactive Multimodal Intervention. This program combined digital education, social platform support, personalized feedback, and nurse-led coaching. The results showed a significant reduction in perceived stress, depression, and sleepiness among healthcare workers. Although physiological parameters (such as heart rate variability) remained unchanged, the intervention still demonstrated the positive psychological impact of flexible and personalized digital tools [106]. Finally, Lu et al. explored the role of confiding as an emotional regulation strategy among shift-working nurses during the COVID-19 pandemic. The intervention, based on reflective writing about received social support, significantly improved interpersonal emotion regulation and, to a lesser extent, sleep quality. However, no significant differences were found in depression levels or intrapersonal emotional regulation, suggesting that the primary benefit stemmed from acknowledging and valuing social support rather than from individual cognitive processing [107]. These studies underscore the effectiveness of personalized, digitally assisted, and relationship-centered interventions in supporting the psychological health of nursing staff. AI, digital platforms, and reflective techniques emerge as promising tools for preventing and treating nurses’ SWDs.

### 4.7. Sleep Education

The literature has increasingly emphasized sleep optimization through strategic napping during night SW. Two recent observational studies by Watanabe et al. confirmed the importance of both nap duration and quality in reducing drowsiness and fatigue. The first longitudinal study showed that naps lasting at least 90 min were associated with less post-nap sleepiness and lower end-of-shift fatigue. However, only 30% of nurses achieved this duration due to environmental factors such as noise and electronic device use. The second study further emphasized the role of sleep quality, revealing that naps with ≥70% sleep efficiency and ≥120 min in bed were the most effective. Individual factors—such as sleep reactivity and pre-nap behaviors like screen use—significantly influenced outcomes [108,109]. These results were supported by the experimental study by Oriyama et al., which assessed the effects of 120 min naps taken at different times during a simulated 16 h night shift. The data indicated that naps between 00:00 and 2:00 offered the greatest benefits in terms of reduced sleepiness and improved cognitive performance in the early morning hours, compared to naps taken at other times. However, increased post-nap sleep inertia highlighted the complexity of nap timing and management in clinical settings [110].

From a behavioral perspective, the qualitative study by Albakri et al. provided valuable insights into the individual sleep strategies adopted by nurses. “Good sleepers” tended to implement structured routines before, during, and after shifts—such as modifying the sleep environment, engaging in physical activity, and avoiding stimulants. In contrast, “poor sleepers” often lacked effective strategies, suggesting the need for personalized educational programs to improve sleep hygiene among nursing staff [109].

The descriptive study by Elif et al. highlighted the significant influence of individual chronotype on the quality of life of shift-working nurses. Specifically, evening-type nurses, more frequently assigned to night shifts, reported lower scores in both the physical and mental components of the Short-Form Health Survey (SF-36) compared to their morning-type or intermediate-type colleagues. These findings underscore the importance of considering chronotype in shift scheduling to promote well-being and prevent psychological distress [114]. Aligned with this approach, the RCT by Booker et al. evaluated the effectiveness of a personalized sleep and SW coaching program versus a control group receiving only nutritional advice. Although no significant differences were observed in sick leave, the intervention group showed marked improvements in insomnia, anxiety, depression, and sleep quality—particularly among those at higher risk for SWDs. These findings highlight the potential benefits of tailored, multidimensional interventions in supporting nurses’ physical and psychological health [111]. This evidence underlines the value of integrated, multi-level interventions, including shift personalization based on chronotype, structured nap planning, sleep education, and psychological support—to mitigate the adverse consequences of SWDs.

### 4.8. Music Therapy and Aromatherapy

In the recent literature, an increasing number of studies have explored the effectiveness of holistic non-pharmacological interventions—such as music therapy, aromatherapy, and animal-assisted interventions—as complementary approaches in promoting the physical and psychological well-being of SW nurses.

Music therapy has proven particularly effective in supporting mental health and sleep among nurses with circadian rhythm sleep disorders. The retrospective study by Wang et al. demonstrated that integrating music therapy with pharmacological treatment using melatonin receptor-2 agonists led to a significant improvement in sleep quality and a reduction in anxiety and depression symptoms, with an overall effectiveness rate of 85.56% compared to 50.56% in the control group [112].

Similarly, the RCT by Zamanifar et al. demonstrated that music therapy—alone or in combination with aromatherapy—significantly reduced anxiety levels among clinical nurses [113]. Lee et al. compared the effects of music therapy and aromatherapy on stress, quality of life, and happiness among shift nurses. Both interventions led to significant improvements compared to the control group, with benefits observed in both subjective well-being and physiological markers. The study employed objective measures such as heart rate variability, demonstrating a positive biological impact [114]. Moreover, the RCT by Nasiri A. et al. showed that the inhalation of rosemary essential oil during night shifts significantly reduced sleepiness and improved alertness, as measured by the KSS and the Epworth Sleepiness Scale [115].

Overall, this evidence supports the use of accessible, free side effects and non-invasive interventions—such as music therapy, aromatherapy, and pet therapy—as effective tools to manage stress, improve emotional balance, enhance sleep quality, and boost alertness among SW nurses.

**Figure 3:**
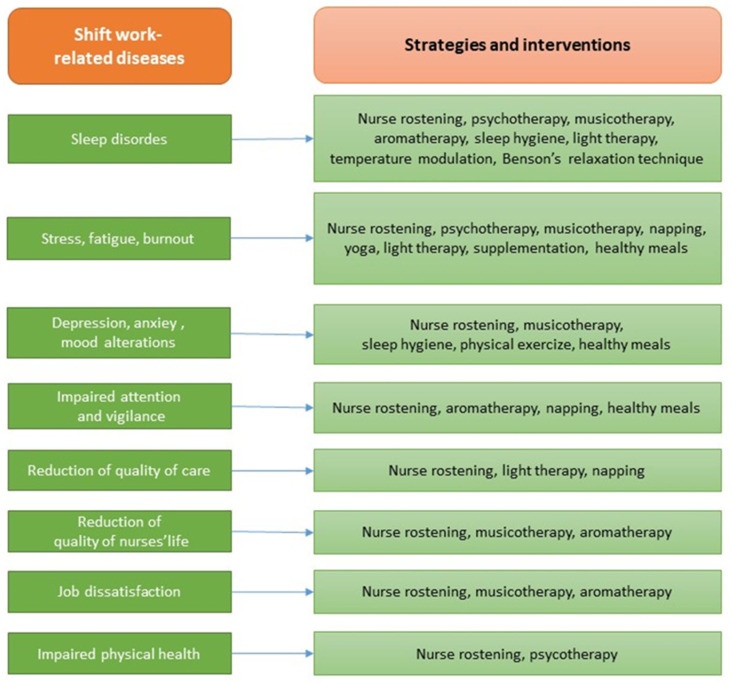
Guidelines for the treatment of nurses’ shift-work-related diseases (SWDs).

Figure 3 correlates the type of nurses’ SWDs with the specific strategies and interventions to prevent or treat it to provide schematic guidelines extrapolated from the reviewed articles.

This study presents several limitations. First, the RCTs reviewed numbered 14 out of 47, the remainder being essentially prospective/observational in nature. Another limitation is the non-representative population, as the sample consists mainly of female or ICU nurses, and there is variation in follow-up duration. Specifically, the most distant evaluation from the intervention was performed after 6 years, whereas the closest evaluation was performed exactly at the end of the intervention. Finally, the authors used different types of validated scales, often in the form of questionnaires or self-reported assessments, which makes it difficult to homogenize the results.

### 4.9. Strengths and Limitations

From a practical perspective, these findings suggest that nursing managers should prioritize the following:

1. Chronotype-aware and flexible scheduling supported by mathematical or digital rostering tools;
2. Structured opportunities for restorative napping;
3. Integration of low-GI nutritional options during night shifts;
4. Access to supervised or digitally supported physical activity programs;
5. Incorporation of evidence-based psychological support programs, potentially AI-assisted;
6. Availability of low-cost, low-risk complementary interventions such as music therapy or aromatherapy.

Future research should focus on large-scale randomized controlled trials and multicenter longitudinal studies to better evaluate the long-term efficacy, cost-effectiveness, and scalability of the most promising strategies—particularly chronotype-based scheduling, timed light exposure, and multimodal digital health interventions. Greater standardization of outcome measures (e.g., validated sleep quality scales, biochemical markers) would enhance comparability across studies.

A key strength of this review is the comprehensive scope, which includes both well-established and emerging interventions, offering a broad overview of current strategies against SWDs. The inclusion of multiple study designs and international samples enhances the generalizability of the observations. However, limitations include the heterogeneity of study designs, small sample sizes, inconsistent follow-up durations, and a predominance of female ICU nurses, which may limit applicability to other settings. The variability in intervention protocols also makes it difficult to determine the relative effectiveness of each strategy.

## 5. Conclusions

SW, especially at night, alters the circadian rhythm, negatively affecting nurses’ mental and physical health, impairing their quality of life, and jeopardizing patient safety.

The interventions or strategies identified to prevent and treat nurses’ SWDs are the following: improved SW planning (nurse rostering); modulation of light and temperature; administration of supplements (e.g., melatonin and zinc); introduction of napping; provision of healthy meals; physical activity, relaxation techniques, and yoga; sleep hygiene education programs; psychotherapy and coaching programs; and, finally, alternative approaches such as music therapy and aromatherapy.

The delivery of such strategies and interventions can be implemented through tailor-made approaches, as well as using mathematical models and digital media. For example, nursing managers could conduct surveys to identify nurses’ individual preferences, tolerance factors, and individual chronotypes and then enter these parameters into dedicated digital applications to ensure satisfactory shifts. It would also be advisable to install light and temperature sensors in departments and invest in educational and psychotherapeutic training programs. In the long run, these investments would save on healthcare costs related to patient risks and employee illnesses.

The parameters most evaluated in the various studies were sleep quality and stress/fatigue level, followed by nurse satisfaction, quality of life, alertness, general mental and physical health, and quality of care.

Further research is mandatory to assess whether the above-mentioned medical, psychotherapeutic, and educational interventions should be systematically performed by nursing managers to improve nurses’ quality of life and quality of care.

## Figures and Tables

**Table 1:** Article screening strategy.

**Article Screening Strategy**	**Keywords: A: “shift work”; B: “nurses”**
	**Boolean Indicators: “A” AND “B”**
	**Timespan: from 1 January 2015 to 3 March 2025**
	**Electronic Databases: PubMed, Scopus, Web of Science, and Cochrane**

**Table 2:** Summary table of interventions and main outcomes.

Intervention on Category	No. of Studies	Study Designs (RCT/Obs./Other)	Main Targeted Outcomes	No. Studies Reporting Improvement
Shift planning and individual factors	12	3 RCT, 9 Obs.	Sleep quality, fatigue, work engagement, absenteeism	10
Satisfaction questionnaires	5	0 RCT, 5 Obs.	Job satisfaction, workload, compliance with ergonomic guidelines	4
Light and temperature modulation	4	2 RCT, 2 Obs.	Sleepiness, fatigue, performance, thermal comfort	3
Supplementation	2	2 RCT	Sleep efficiency, latency, micronutrient status	2
Meal management	3	2 RCT, 1 Obs.	Glycemic control, mood, metabolic parameters	3
Physical activity, relaxation, yoga	3	2 RCT, 1 Exp.	Aerobic capacity, depression, sleep quality	3
Psychotherapy	4	3 RCT, 1 Non-RCT	Burnout, stress, insomnia	4
Sleep education	6	2 RCT, 4 Obs.	Fatigue, sleep quality, anxiety	5
Music and aromatherapy	4	3 RCT, 1 Retrospective	Sleep quality, anxiety, stress, alertness	4
Total	43	—	—	—

**Table 3:** Overall risk of bias assessment in studies: analysis based on ROBINS-I.

Study	Bias Due to Confounding	Selection Bias	Bias in Classification of Interventions	Bias Due to Deviations from Intended Interventions	Bias Due to Missing Data	Bias in Measurement of Outcomes	Bias in Selection of Reported Results	Overall Risk of Bias
Niu S.F. et al. (2015) [77]	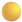	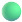	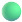	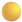	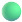	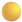	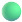	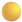
Schiffer D. et al. (2018) [78]	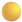	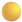	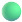	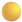	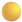	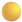	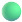	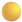
Dall’Ora C. et al. (2020) [79]	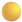	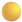	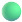	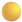	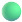	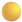	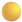	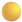
Inoue M. et al. (2020) [80]	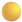	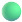	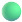	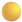	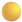	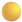	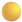	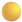
Waage S. et al. (2021) [81]	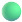	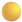	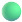	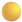	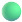	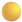	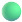	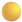
Kubo T. et al. (2022) [82]	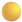	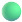	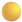	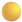	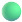	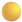	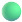	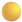
Jung H.S et al. (2015) [83]	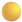	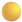	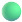	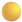	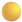	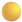	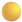	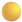
Pahlevanzadeh M.J. et al. (2021) [84]	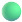	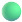	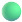	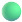	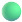	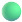	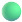	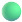
Bülbül E. et al. (2023) [85]	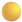	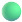	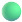	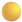	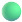	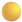	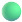	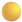
Li J.N et al. (2023) [86]	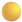	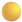	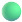	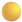	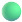	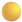	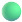	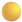
de Bruijn L. et al. (2024) [87]	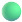	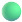	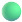	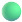	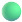	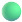	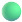	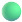
Dehring T. et al. (2018) [88]	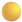	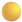	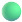	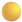	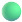	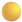	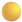	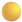
Abed Al Ahad M. et al. (2021) [89]	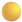	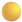	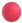	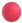	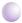	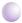	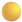	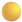
Lee J. et al. (2021) [90]	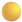	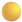	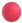	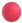	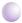	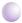	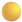	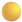
Booker L.A. et al. (2024) [91]	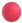	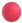	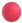	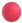	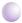	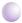	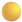	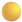
Shin S.H. et al. (2024) [92]	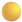	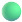	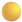	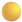	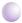	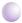	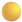	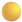
Griepentrog J.E. et al. (2018) [93]	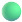	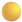	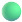	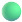	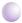	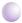	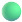	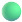
Bjorvatn B. et al. (2021) [94]	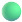	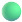	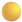	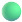	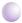	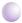	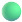	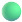
Hoshi H. et al. (2022) [95]	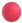	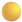	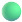	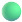	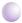	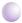	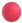	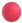
Kim J.H. et al. (2020) [96]	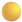	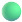	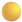	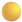	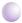	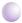	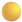	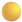
Sadeghniiat-Haghighi et al. (2016) [97]	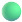	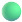	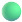	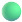	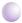	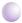	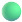	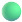
Gholipour Baradari A. et al. (2017) [98]	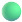	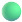	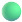	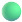	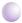	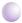	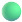	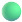
Suyoto et al. (2024) [99]	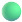	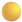	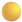	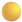	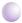	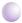	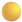	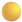
Leedo E. (2017) [100]	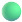	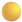	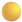	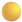	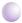	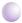	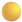	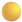
Matsugaki R. (2017) [101]	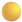	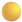	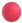	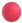	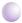	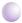	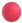	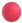
Baek Y. (2022) [102]	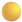	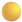	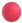	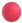	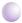	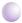	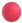	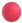
Yoko M. (2019) [103]	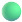	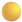	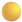	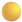	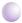	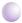	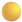	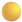
Baek G. et al. (2025) [104]	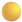	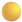	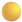	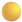	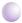	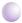	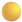	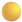
Eli et al. (2024) [105]	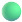	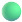	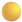	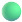	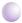	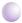	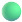	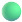
Wenhua Lu et al. (2024) [106]	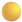	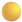	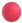	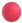	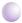	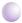	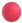	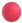
Cui Lu et al. (2025) [107]	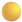	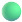	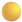	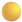	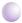	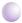	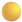	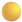
Elif B. et al. (2023) [85]	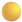	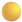	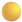	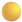	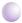	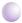	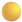	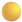
Watanabe K. et al. (2022) [108]	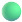	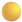	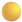	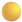	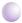	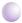	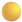	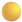
Watanabe K. et al. (2025) [109]	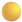	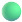	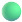	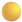	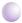	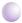	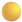	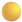
Oriyama S. et al. (2019) [110]	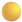	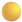	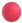	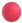	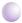	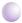	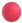	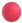
Albakri U. et al. (2023) [109]	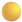	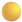	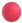	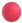	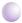	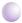	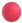	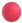
Booker L.A. et al. (2022) [111]	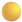	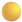	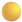	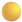	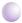	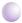	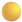	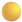
Wang X. et al. (2024) [112]	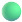	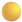	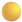	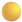	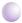	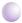	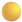	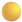
Zamanifar et al. (2020) [113]	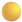	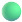	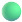	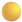	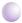	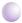	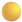	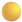
Lee Sh. et al. (2024) [114]	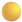	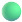	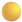	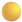	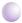	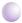	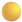	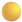
Nasiri A. et al. (2021) [115]	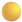	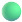	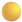	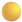	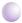	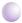	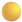	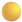
Molzof et al. (2017) [116]	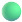	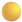	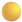	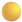	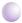	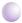	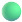	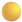

Legend: 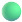 low risk; 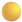 moderate risk; 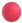 high risk; 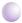 missing data.

**Table 4:** Studies concerning the effects of work schedules, flexibility, and recovery time on nurses’ shift-work-related diseases (SWDs).

Authors	Study Design	Study Sample (N. Nurses)	Interventions/ Strategies	Follow-Up	Parameters Evaluated	Outcomes
Niu S.F. et al. (2015) [77]	Prospective longitudinal, parallel group, comparative	63 randomly assigned to night and day-shift groups	Salivary cortisol measurement to assess circadian secretion patterns	Observation over work shifts and recovery periods	Circadian cortisol levels, cortisol awakening response, SW impact	Night-shift nurses required at least 4 days to adjust their circadian rhythm; 2 days off were necessary to restore diurnal cortisol levels. Night shifts led to altered cortisol profiles, affecting adaptation and recovery.
Schiffer D. et al. (2018) [78]	Cross-sectional	100 females in Northern Italy	Questionnaire and daily diary assessing sleep, work performance, and work–life balance.	None	Sleep quantity/quality, work performance, work–life balance	CW shift nurses had better sleep quality, fewer awakenings, and better work–life balance than CCW shift nurses. CCW rotation was linked to more sleep disturbances, attention deficits, and social/family life interference.
Dall’Ora C. et al. (2020) [79]	Cross-sectional survey	31,627 from 487 hospitals in 12 European countries	Comparison of 12 h shifts vs. shorter shifts in relation to education, discussion opportunities, continuity of care, and information loss during handovers.	None	Participation in continuing education programs, time for discussion with colleagues, continuity of care, and loss of patient information during handovers	Long shifts were associated with reduced educational opportunities and fewer discussions on patient care. No significant association with continuity of care or information loss was found.
Inoue, M. et al. (2020) [80]	Survey-based observational	805 in Japan	Evaluation of a variable shift system and break activities.	Self-reported survey data analysis	Work engagement, stress, fatigue, break activities	Variable shift system supports work engagement; workload correlates with stress; effective breaks improve engagement.
Waage S. et al. (2021) [81]	Longitudinal cohort	1076	Changes in work schedules, reduction/increase in night shifts, and quick returns.	2 yrs.	SWDs prevalence, number of night shifts, number of quick returns	Stopping night shifts reduced SWD prevalence; increasing night shifts increased SWD prevalence.
Kubo T. et al. (2022) [82]	Non-randomized controlled cross-over	30 females in Japan	Extended restart breaks from 31 h to 55 h after consecutive night shifts, subjective and objective fatigue, and sleep measurements	5-mo.	Vital exhaustion, psychological distress, hair cortisol, salivary C-reactive protein, sleep patterns	Extended restart breaks moderately reduced fatigue and distress. No significant effect on objectively measured stress, sleep, or biomarkers

Note: CCW, counterclockwise; CW, clockwise; mo., months; SW, shift work; SWDs, shift-work-related diseases; yrs., years.

**Table 5:** Studies concerning the effects of individual tolerance factors and chronotype on nurses’ shift-work-related diseases (SWDs).

Authors	Study Design	Study Sample (N. Nurses)	Interventions/ Strategies	Follow-Up	Parameters Evaluated	Outcomes
Jung H.S. et al. (2015) [83]	Cross-sectional, correlational	660 females in South Korea	Questionnaire on demographics, lifestyle, and work conditions	None	SWT, insomnia, fatigue, depression	SWT influenced by self-esteem, job stress, morningness, and physical activity. Job stress was the key factor. Physical activity reduced insomnia and fatigue, while alcohol increased fatigue. More depression in younger nurses
Pahlevanzadeh M.J. et al. (2021) [84]	Mathematical modeling, real case	Mathematical modeling, real case: 18 male nurses, 11 female nurses without a child, 7 female nurses with a child	Binary integer programming model for nurse scheduling using Z-number method for justice-based shift assignment	Performance assessed post-implementation	Nurse attendance, complaints, and satisfaction	40% reduction in absences, 50% reduction in complaints, 30% increase in satisfaction
Bülbül E. et al. (2023) [85]	Descriptive	267 females	MEQ, SF-36	None	Chronotypes of nurses, quality of life, number of night shifts, sleep patterns	Evening-type nurses had lower quality of life; morning-type nurses were older and had more work experience; evening-type nurses had more night shifts and lower scores in physical and mental health
Li J.N. et al. (2023) [86]	Multicenter cross-sectional	1426 female MCH in China	Demographic questionnaire, self-reported chronotype, PSQI, WRQOL-2 scale.	None	Sleep quality, chronotype, night shift schedule, quality of work life	57.9% of nurses had poor sleep quality. Chronotype and work–life quality were major predictors. Night shift schedule had no significant effect on adjusted models. Poor sleep was linked to older age, caffeine intake, and irregular meals
de Bruijn L. et al. (2024) [87]	Cohort	37,731 Dutch females	Self-reported assessments of SW history, shift type preference, chronotype classification, MOS-SPI-II	6 yrs.	Chronotype stability, sleep timing in work-free periods, shift type preference, sleep problems related to SW	Evening types preferred night shifts, while morning types favored day shifts. Intermediate chronotypes had fewer sleep problems compared to morning or evening types. Chronotype remained stable over six years, with gradual shifts towards morningness. Extreme chronotypes experienced greater circadian disruption, affecting sleep quality and SWT

Note: MCH, maternal and child health; MEQ, morningness–eveningness questionnaire; MOS-SPI-II, sleep–wake timing and sleep problems; PSQI, Pittsburgh sleep quality index; SW, shift work; SWT, shift work tolerance; SF-36, short-form health survey; WRQOL-2, work-related quality of life-2; yrs., years.

**Table 6:** Studies concerning the effects of satisfaction questionnaires on nurses’ shift-work-related diseases (SWDs).

Authors (Year)	Study Design	Study Sample (N. Nurses)	Interventions/ Strategies	Follow-Up	Parameters Evaluated	Outcomes
Dehring T. et al. (2018) [88]	Cross-sectional	108 registered nurses from two Melbourne health services. 98 females	Survey on demographic characteristics, organizational climate, and health outcomes	None	Organizational climate factors, health outcomes (general health, social dysfunction, stress)	Rotating shift nurses had higher coworker cohesion; night staff reported greater physical comfort. Supervisor support predicted better health outcomes. Task orientation reduced social dysfunction. Enhancing organizational climate could mitigate negative health effects of SW
Abed Al Ahad M. et al. (2021) [89]	Longitudinal	90 female registered nurses, 1303 responses, Lebanese hospital	Daily surveys assessing work satisfaction, workload, patient-to-nurse ratio, and rationed care	day 91	Shift-specific workload, job satisfaction, implicit rationing of care	Work satisfaction varied among individual nurses but not across units. Lower workload and less rationed care improved satisfaction. Objective workload (patient-to-nurse ratio) was not a strong predictor. Improving scheduling and teamwork may enhance satisfaction
Lee J. et al. (2021) [90]	Descriptive	182 females working in three shifts for more than one year in superior general and general hospitals:	Self-administered questionnaire, 13-week work schedule tables analyzed based on 17 work schedule recommendations (WSRs), person-based and cycle-based compliance assessments	None	General nurse characteristics, compliance with 17 WSRs, factors affecting compliance (hospital type, workplace standards, nurses in school/pregnancy)	Compliance with WSRs averaged 11.77/17. No nurse fully adhered to “no work on weekends.” Factors like hospital type, institutional standards, and demographics influenced compliance. Frequent weekend work and consecutive night shifts led to inadequate rest. Two-day rest after night shifts was rarely followed. Hospitals with scheduling standards showed better compliance
Booker L.A. et al. (2024) [91]	Qualitative single case	24 across 3 hospitals in Victoria, Australia	Semi-structured interviews, thematic analysis, grounded theory methods	None	Rostering process, training, fatigue risks, SW practices, cultural barriers	Rostering staff lacked formal training, self-rostering was common but led to fatigue concerns, cultural resistance to change, and the need for better education on safe shift scheduling
Shin S.H. et al. (2024) [92]	Methodological	247 in Korea	Literature review, in-depth interviews, expert validation, questionnaire testing	None	QHWE, reliability, validity, job satisfaction	Developed a 23-item instrument measuring physical, psychological, social, and structural factors. High reliability and validity confirmed. Can assess and improve nurses’ work environment

Note: QHWE, quality of healthy work environment; SW, shift work; WSRs, work schedule recommendations.

**Table 7:** Studies concerning the effects of light and temperature modulation on nurses’ shift-work-related diseases (SWDs).

Authors (Year)	Study Design	Study Sample (N. Nurses)	Interventions/ Strategies	Follow-Up	Parameters Evaluated	Outcomes
Griepentrog J.E. et al. (2018) [93]	Randomized, crossover	43 matched pairs of ICU (31 subjects + 12 in both phases); 71% female	High illuminance light (1500–2000 lx) for 10 h during night shift Compared with standard hospital lighting (300 lx)	Each nurse completed both lighting exposures; primary assessments were performed at 5:00 h (end of exposure)	SSS PVT: errors, lapses, reaction time Salivary melatonin levels	Reduced sleepiness under bright light. Increased psychomotor errors. No significant changes in PVT lapses or reaction times. Melatonin suppressed more under bright light, but not statistically significant
Bjorvatn B. et al. (2021) [94]	RCT	35	Bright light (10,000 lx) vs. red dim light (100 lx) for 30 min at night (timed from 2:00 to 4:00 over 3 nights) Three-night shifts with each light condition	9-day protocol: 3 days before, 3 night shifts, 3 days after; crossover with ≥3-we. washout	Subjective KSS. Accumulated Time with Sleepiness (heavy eyelids, reduced performance). Objective performance PVT. Mood, caffeine intake, general functioning.	Heavy eyelids reduced with bright light on nights 1 and 2. No significant effect on KSS or PVT. No difference in overall functioning after night shifts. Bright light did not impair readaptation to day rhythm
Hoshi H. et al. (2022) [95]	Non-randomized, open-label, quasi-experimental	20 females (17 analyzed for dark; 10 for well-lit condition):	Comparison of dark lighting (110 lx) vs. bright lighting (410 lx) at nurse workstations during night shifts	Two lighting phases: Nov–Dec 2015 (dark); Jan–Feb 2016 (well-lit)	Subjective fatigue and sleepiness (instability, uneasiness, grogginess, lethargy, drowsiness). Sleep quality (sleepiness on waking, sleep induction/maintenance, dreaming, recovery from fatigue, sleep duration). Incident/accident reports.	Fatigue and sleepiness were higher under dark lighting (e.g., lethargy and drowsiness significantly increased) No significant difference in sleep quality No negative impact on work performance (incident/accident rates not significantly affected)
Kim J.H. et al. (2020) [96]	Crossover	20 female (2 groups of 10)	Nurses worked in two temperature-controlled environments: 23 °C CTE 26 °C NTE Each nurse experienced both conditions for 2 consecutive night shifts.	2-night shifts per condition, with 2-we. washout between	Thermal sensation Night work tolerance (fatigue, sleepiness, adaptation) Body temperature Urinary 6-sulphatoxymelatonin	23 °C improved thermal comfort. Body temperature was lower at 23 °C. Melatonin significantly decreased on the 2nd night in 23 °C group. No significant difference in fatigue, sleepiness, or adaptation scores between conditions

Note: CTE, compensated temperature environment; ICU, intensive care unit; KSS, Karolinska sleepiness scale; NTE, natural temperature environment; PVT, psychomotor vigilance test; SSS, Stanford sleepiness scale; we., weeks.

**Table 8:** Studies concerning the effects of supplementation on nurses’ shift-work-related diseases (SWDs).

Authors (Year)	Study Design	Study Sample (N. Nurses)	Interventions/ Strategies	Follow-Up	Parameters Evaluated	Outcomes
Sadeghniiat-Haghighi et al., (2016) [97]	RCT	39 males with difficulty falling asleep (from 50 enrolled, out of 295 screened).	3 mg melatonin vs. placebo taken 30 min. before sleep for 3 nights, with a 2 we. washout period	3-night intervention periods for both melatonin and placebo	TST SE SOL WASO	SE increased with melatonin (82.2% → 85.5%) SOL decreased (0.27 h → 0.20 h) No significant change in TST and WASO No adverse effects reported.
Gholipour Baradari A. et al. (2017) [98]	RCT	53 female ICU nurses (27 zinc group, 26 placebo group).	Zinc sulfate capsules (220 mg) every 72 h for 1 mo. vs. placebo	1 mo.	Sleep Quality (PSQI: total and 7 components) Serum Zinc and Copper levels	Improved total sleep quality score with zinc. Significant improvement in subjective sleep quality and sleep latency. Serum zinc levels increased significantly in zinc group. No adverse effects reported.

Note: Mo., months; SE, sleep efficiency; SOL, sleep onset latency; TST, total sleep time; PSQI, Pittsburgh sleep quality index; WASO, wakening after sleep onset; we., weeks.

**Table 9:** Studies concerning the effects of meal management on nurses’ shift-work-related diseases (SWDs).

Authors (Year)	Study Design	Study Sample (N. Nurses)	Interventions/ Strategies	Follow-Up	Parameters Evaluated	Outcomes
Suyoto et al. (2024) [99]	RCT	49	Comparison of night-shift meal strategies: no meal, 1 high-GI meal, 1 low-GI meal, 3 high-GI meals, 3 low-GI meals	3 intervention periods: 3 days each with 2 we. of washout	CGM, AUCmin, PEAK, MEAN, CV, GVP, CONGA1h	1 or 3 high-GI meals increased glycemic variability and peak glucose levels; 1 or 3 low-GI meals had no significant effect on glycemic control or variability vs. fasting; meal frequency had no independent effect;
Molzof et al. (2017) [116]	Observational, comparative	17	9-day food intake recording, evaluation of inflammatory potential of diet (Dietary Inflammatory Index™), fasting metabolic panel on day off	9 days + 1 fasting blood sample	Dietary intake by shift and time of day; CMS risk factors (lipids, HDL, etc.); inflammatory potential	Night-shift food intake (total grams) was positively associated with lipid levels; daytime intake was more pro-inflammatory for all nurses, regardless of shift
Leedo E. (2017) [100]	RCT	60	Healthy cold lunch, healthy snack, and water vs. usual diet (control)	Start point; end point	Reaction time (Go/No-Go test) Mood (POMS) Food intake (food diary 4 days)	No effect on reaction time. In shift workers: ↓ fatigue, ↑ vitality, ↓ mood disturbance ↑ intake of water, carbohydrates, fiber; ↓ fat

Note: AUCmin, glycemic control; CGM, continuous glucose monitoring; CV, variability coefficient; GVP, glycemic variability percentage; CONGA1h, continuous overall net glycemic action (1 h); GI, glycemic index; Go/No-Go test, reaction time; MEAN, glycemic control; PEAK, glycemic control; POMS, profile of mood states.

**Table 10:** Studies concerning the effects of physical activity, relaxation techniques, and yoga on nurses’ shift-work-related diseases (SWDs).

Authors (Year)	Study Design	Study Sample (N. Nurses)	Interventions/ Strategies	Follow-Up	Parameters Evaluated	Outcomes
Matsugaki, R. (2017) [101]	RCT	30 SG: 15 VG: 15	SG: Exercises under the supervision of a PT, twice a we. for 12 we, including aerobic and resistance training. VG: Exercises without supervision, with instruction only in the first session and encouragement by e-mail biweekly.	12 we.	Aerobic capacity (VO2 max) Muscle strength Anthropometric data (BMI, muscle mass, fat mass) Biochemical parameters (total cholesterol, HDL, LDL, triglycerides, blood glucose, insulin, high molecular weight adiponectin, reactive oxygen metabolites) Mental health (depression levels, mood status with POMS)	Exercise supervised by a physical therapist has been shown to be more effective than voluntary exercise alone in improving aerobic capacity, muscle strength, and certain biochemical parameters
Baek Y. (2022) [102]	experimental study	54 (I.G.: 25; C.G.: 24)	Smartphone-based home workout program; Text-message counseling; Environmental improvement	18 we.	Sleep disturbance, fatigue, musculoskeletal problems, resilience, and nursing performance	The I.G. showed significant improvements
Yoko M. (2019) [103]	Randomized crossover trial	20	Restorative Yoga Program; Control Period; crossover	9 we.	Psychological and physical stress Vital signs (blood pressure, heart rate). Body weight	Yoga significantly reduced psychological and physical stress reactions No significant changes in vital signs and body weight were observed. A reduction in stress after 4 weeks of home practice, with more pronounced effects than a single guided session

Note: BMI, body mass index; HDL, high-density lipoprotein; LDL, low-density lipoprotein; PT, physical therapist; SV, supervised group; VG, voluntary group; we., weeks.

**Table 11:** Studies concerning the effects of physiotherapy on nurses’ shift-work-related diseases (SWDs).

Authors (Year)	Study Design	Study Sample (N. Nurses)	Interventions/ Strategies	Follow-Up	Parameters Evaluated	Outcomes
Baek G. et al. (2025) [104]	RCT	120	E.G.: AI-assisted tailored intervention, selecting from four programs C.G.1: self-selected one of the four programs. C.G.2: online information on burnout reduction.	4 we., with assessments at baseline, we. 2, and we. 4	P. O.: client-related burnout, personal burnout, and work-related burnout. S.O.: job stress, stress responses, and coping strategies	E.G.: significant reductions in client-related burnout and personal burnout; Stress response reduction highest in C.G.1 N.S.D. for work-related burnout and job stress reduction
Eli et al. (2024) [105]	RCT	120	dCBT-I	6 mo.	Insomnia severity index, sleep efficiency, daytime functioning, and psychological distress	dCBT-I group showed significant reductions in insomnia severity and psychological distress, with improvements in sleep efficiency and daytime functioning compared to the control group.
Wenhua Lu et al. (2024) [106]	Non-RCT	245	I.G.: CIMI, consisting of mobile stress management education, a web-based WeChat social network, personalized feedback, and support from a coach nurse. C.G.: self-guided stress management intervention.	12 we.	Psychological indicators (perceived stress, mental distress, subjective happiness), physiological indicators (heart rate variability), and sleep-related measures (fatigue and sleepiness)	CIMI reduced perceived stress, depression, fatigue, and sleepiness while increasing subjective happiness. N.S.D. in physiological stress indicators were observed between the groups
Cui Lu et al. (2025) [107]	RCT	66 I.G.: 34 C.G.: 32	I.G.: online intervention focused on reflecting on social support obtained from confiding about work-related hassles C.G.: recorded confiding activities twice a week without reflecting on social support	8 we.	IERQ, SRGHSQ and PHQ-9	The confiding intervention enhanced interpersonal emotion regulation but had limited effects on intrapersonal emotion regulation and well-being

Note: C.G., control group; CIMI, interventional multimodal interactive complex; dCBT-I, digital cognitive behavioral therapy for insomnia; E.G., experimental group; ERQ, intrapersonal emotion regulation: cognitive reappraisal, expressive inhibition; I.G., intervention group; AI: artificial intelligence; C.G.: control group; CIMI: interactive multimodal intervention; dCBT-I, digital cognitive behavioral therapy for insomnia; IERQ, interpersonal emotion regulation; I.G., intervention group; mo., months; N.S.D., no significant differences; P.O, primary outcomes; PHQ-9, depression; SRGHSQ, self-rated general health and sleep quality; S.O, secondary outcomes; we., weeks.

**Table 12:** Studies concerning the effects of sleep education on nurses’ shift-work-related diseases (SWDs).

Authors (Year)	Study Design	Study Sample (N. Nurses)	Interventions/ Strategies	Follow-Up	Parameters Evaluated	Outcomes
Elif B. et al. (2023) [85]	Descriptive, cross-sectional	267	No clinical intervention; assessment through questionnaires: MEQ and SF-36	None	Chronotype (morning, intermediate, evening via MEQ) Health-related quality of life (via SF-36)	68.9% intermediate-type, 15.7% morning-type, 15.4% evening-type Evening-type nurses had more night shifts and significantly lower scores in physical, mental, and social domains of SF-36
Watanabe K. et al. (2022) [108]	Longitudinal observational	49	No intervention; effects of natural 90 min napping during 16 h night shifts	4-night shifts per nurse over 1 mo.	Total nap duration (TND) Environmental factors (noise, light, bedding, etc.) Fatigue levels Use of electronics, nap break duration, activity levels	Longer naps (≥90 min) are linked to less post-nap drowsiness and reduced fatigue at shift end Environmental factors (noise, screen time, nap time length) significantly influenced ability to nap ≥90 min More experienced nurses were more likely to achieve ≥90 min naps
Watanabe K. et al. (2025) [109]	Prospective observational	32	No clinical intervention; measured natural nap behaviors using activity monitors and surveys	1 mo. (data collected from 120-night shifts; 105 included in nap-related analysis)	Nap quantity: TIB Nap quality: SE Fatigue Sleepiness Individual and environmental factors	TIB ≥ 120 min and SE ≥ 70% were optimal for reducing fatigue and sleepiness Low SE or short naps increased post-shift fatigue Factors influencing nap success: sleep reactivity, electronic device use, and prophylactic naps
Albakri U. et al. (2023) [109]	Qualitative descriptive study using semi-structured interviews	34 (17 good sleepers, 17 poor sleepers) working irregular night shifts	No formal intervention: participants discussed strategies for sleep before, during, and after night shifts	None	Sleep quality Strategies used for sleep and alertness management Lifestyle and behavioral routines	Sleep quality Strategies used for sleep and alertness management Lifestyle and behavioral routines
Booker, L.A. et al. (2022) [111]	RCT	149	I.G.: 8-week individualized sleep and SW education + coaching program C.G.: Coaching on low glycemic index dietary habits	6 mo. (pre- and post-intervention sick leave analysis + questionnaires at baseline/follow-up)	ISI SHI PHQ-9 GAD-7 FOSQ-1 0SWD risk	No significant difference in sick leave reduction Improvements in insomnia and depression in both groups Anxiety and sleep function improved significantly only in the intervention group
Oriyama, S. et al. (2019) [110]	Pilot crossover experimental	14	120 min. naps at 3 different times: 22:00–00:00 (22-NAP) 00:00–02:00 (00-NAP) 02:00–04:00 (02-NAP)	3 repeated lab experiments per participant, spaced 1 month apart	Sleep metrics: TST, SE, SOL, WASO Cognitive performance (mental arithmetic) VAS Sublingual temperature Heart rate variability	Sleep parameters are similar across all naps post-nap: ↑ sleepiness, fatigue, ↓ performance 00-NAP showed better early-morning outcomes 02-NAP showed highest fatigue immediately after nap

Note: FOSQ-10, functional sleep outcomes; GAD-7: anxiety; ISI, insomnia; MEQ, morningness–eveningness questionnaire; min., minutes; mo., months; NAP, napping; PHQ-9, depression; SE, sleep efficiency; SF-36, short-form health survey; SHI, sleep hygiene; SOL, sleep onset latency; SW, shift work; TIB, time in bed; TND, total nap duration; TST, total sleep time; VAS, subjective sleepiness and fatigue; WASO, wake after sleep onset; 22-NAP, 22:00–00:00; 00-NAP, 00:00–2:00; 02-NAP, 2:00–4:00.

**Table 13:** Studies concerning the effects of music therapy and aromatherapy on nurses’ shift-work-related diseases (SWDs).

Authors (Year)	Study Design	Study Sample (N. Nurses)	Interventions/ Strategies	Follow-Up	Parameters Evaluated	Outcomes
Wang X. et al. (2024) [112]	Retrospective	360 I.G.:180 C.G.: 180	I.G.: MT2 treatment + music therapy C.G.: MT2 treatment.	Six treatment courses, with evaluations before and after.	SAS, SDS, PSQI, CE	I.G.: SAS, SDS, PSQI, and CE significantly improved
Lee Sh. et al. (2024) [114]	RCT	78	M.G.: Listened to music for at least 30 min, three times a day, for one week A.G.: Continuous inhalation of a blend of essential oils (lavender, ylang-ylang, and lemon) through an aroma necklace worn for one week. C.G.: no intervention	One we., with pre- and post-treatment evaluations.	LK, SI, QOL, OHQ	Music therapy and aromatherapy are effective in reducing stress and improving quality of life
Zamanifar et al. (2020) [113]	RCT	120	I.G.: (1) music therapy, (2) aromatherapy with chamomile-lavender oil, (3) both music therapy and aromatherapy C.G.: no intervention	After 3 consecutive work shifts	BAI	Significant reduction in anxiety in all three groups. Aromatherapy group had the lowest anxiety score post-intervention.
Nasiri A. et al. (2021) [115]	RCT	80 I.G.:40 C.G.:40	I.G.: inhalation of one drop of rosemary essential oil via mask C.G.: inhalation of distilled water during night shift for 2 h	Before and after intervention during the same shift	KSS, ESS	Rosemary oil significantly decreased sleepiness and increased alertness

Note: A.T., aromatherapy group; BAI, Beck anxiety inventory; CE, clinical efficacy; ESS, Epworth sleepiness scale; KSS, Karolinska sleepiness scale; LK, perceived stress; M.G., musicotherapy group; MT2, melatonin receptor agonist; OHQ, Oxford happiness questionnaire; PSQI, Pittsburgh sleep quality index scale; QOL, quality of life; SAS, self-assessment anxiety scale; SDS, self-rating depression scale; SI, stress index; VAS, visual analog scale; we., week.

## Data Availability

Not applicable. All figures are produced by the authors.

## Abbreviations

AI	Artificial Intelligence
CHD	Coronary Heart Disease
CI	Confidence Interval
COVID-19	Coronavirus Disease 2019
CVD	Cardiovascular Disease
DNA	Deoxyribonucleic Acid
GI	Glycemic Index
KSS	Karolinska Sleepiness Scale
ICU	Intensive Care Unit
MetS	Metabolic Syndrome
MS	Multiple Sclerosis
RCT	Randomized Controlled Trial
SW	Shift Work
SWD	Shift-Work-Related Diseases

## References

[1] Rosa D., Terzoni S., Dellafiore F., Destrebecq A. (2019). Systematic Review of Shift Work and Nurses’ Health. Occup. Med..

[2] Imes C.C., Barthel N.J., Chasens E.R., Dunbar-Jacob J., Engberg S.J., Feeley C.A., Fennimore L.A., Godzik C.M., Klem M.L., Luyster F.S. (2023). Shift Work Organization on Nurse Injuries: A Scoping Review. Int. J. Nurs. Stud..

[3] Jørgensen J.T., Karlsen S., Stayner L., Hansen J., Andersen Z.J. (2017). Shift Work and Overall and Cause-Specific Mortality in the Danish Nurse Cohort. Scand. J. Work. Environ. Health.

[4] Min A., Hong H.C., Kim Y.M. (2022). Work Schedule Characteristics and Occupational Fatigue/Recovery among Rotating-Shift Nurses: A Cross-Sectional Study. J. Nurs. Manag..

[5] Di Muzio M., Dionisi S., Di Simone E., Cianfrocca C., Di Muzio F., Fabbian F., Barbiero G., Tartaglini D., Giannetta N. (2019). Can Nurses’ Shift Work Jeopardize the Patient Safety? A Systematic Review. Eur. Rev. Med. Pharmacol. Sci..

[6] Cooper P.G. (2008). A Call for a Paradigm Shift in Nursing and Healthcare Leadership. Nurs. Forum.

[7] Barton J., Spelten E.R., Smith L.R., Totterdell P.A., Folkard S. (1993). A Classification of Nursing and Midwifery Shift Systems. Int. J. Nurs. Stud..

[8] White E. (2017). A Comparison of Nursing Education and Workforce Planning Initiatives in the United States and England. Policy Politics Nurs. Pract..

[9] Beckman R.J., Hutton S., Czekanski E., Vance K., Mohr D.C. (2022). A Comparison of Shift Length and Nursing and Quality Outcomes in Acute Inpatient Mental Health Units. J. Nurs. Adm..

[10] Niu S.-F., Chu H., Chen C.-H., Chung M.-H., Chang Y.-S., Liao Y.-M., Chou K.-R. (2013). A Comparison of the Effects of Fixed- and Rotating-Shift Schedules on Nursing Staff Attention Levels: A Randomized Trial. Biol. Res. Nurs..

[11] Ashraf H., Bodapati A., Hanif A., Okafor D.K., Katyal G., Kaur G., Khan S. (2023). Safety and Efficacy of Biologic Therapies (Ustekinumab and Vedolizumab) in the Treatment of Inflammatory Bowel Disease (IBD): A Systematic Review. Cureus.

[12] Kivimäki M., Virtanen M., Elovainio M., Väänänen A., Keltikangas-Järvinen L., Vahtera J. (2006). Prevalent Cardiovascular Disease, Risk Factors and Selection out of Shift Work. Scand. J. Work. Environ. Health.

[13] Feng T., Booth B.M., Baldwin-Rodríguez B., Osorno F., Narayanan S. (2021). A Multimodal Analysis of Physical Activity, Sleep, and Work Shift in Nurses with Wearable Sensor Data. Sci. Rep..

[14] Seo Y.-E., Kim T.-Y., Yoo H.-S., Chae M.S. (2022). A Postanaesthesia Workload Instrument Can Provide Objective Information Promoting Appropriate Workload Distribution between Day and Evening Shift Nursing Staff in the Postanaesthesia Care Unit: A Prospective Observational Cohort Study. Eur. J. Anaesthesiol..

[15] Inchingolo F., Tatullo M., Abenavoli F.M., Marrelli M., Inchingolo A.D., Corelli R., Inchingolo A.M., Dipalma G. (2011). Surgical treatment of depressed scar: A simple technique. Int. J. Med. Sci..

[16] Manias E., Aitken R., Peerson A., Parker J., Wong K. (2003). Agency-Nursing Work: Perceptions and Experiences of Agency Nurses. Int. J. Nurs. Stud..

[17] Lim S., Han K., Cho H., Baek H. (2019). Shift-Work Nurses’ Work Environments and Health-Promoting Behaviours in Relation to Sleep Disturbance: A Cross-Sectional Secondary Data Analysis. J. Clin. Nurs..

[18] Silva R.M.d., Zeitoune R.C.G., Lenz F.C.D., Pretto C.R., Santos K.M.D., Magnago T.S.B.d.S., Centenaro A.P.F.C. (2024). Sleep Duration and Quality of Brazilian Nursing Staff Who Work in Shifts. Rev. Bras. Enferm..

[19] Niu S.-F., Miao N.-F., Liao Y.-M., Chi M.-J., Chung M.-H., Chou K.-R. (2017). Sleep Quality Associated with Different Work Schedules: A Longitudinal Study of Nursing Staff. Biol. Res. Nurs..

[20] Buja A., Zampieron A., Mastrangelo G., Petean M., Vinelli A., Cerne D., Baldo V. (2013). Strain and Health Implications of Nurses’ Shift Work. Int. J. Occup. Med. Environ. Health.

[21] Wakui T. (2000). Study on Work Load of Matrons under Shift Work in a Special Nursing Home for the Elderly. Ind. Health.

[22] Congdon J., Craft J., Christensen M. (2020). Are We Measuring Nursing Workflow Correctly? A Literature Review. Br. J. Nurs..

[23] Bae S.-H., Fabry D. (2014). Assessing the Relationships between Nurse Work Hours/Overtime and Nurse and Patient Outcomes: Systematic Literature Review. Nurs. Outlook.

[24] Buss J. (2012). Associations between Obesity and Stress and Shift Work among Nurses. Workplace Health Saf..

[25] Giorgi F., Mattei A., Notarnicola I., Petrucci C., Lancia L. (2018). Can Sleep Quality and Burnout Affect the Job Performance of Shift-Work Nurses? A Hospital Cross-Sectional Study. J. Adv. Nurs..

[26] Morse L., Duncan H., Apen L.V., Reese K., Crawford C.L. (2024). Centralized Scheduling of Nursing Staff: A Rapid Review of the Literature. Nurs. Adm. Q..

[27] Inchingolo F., Tatullo M., Abenavoli F.M., Marrelli M., Inchingolo A.D., Servili A., Inchingolo A.M., Dipalma G. (2010). A Hypothetical Correlation between Hyaluronic Acid Gel and Development of Cutaneous Metaplastic Synovial Cyst. Head Face Med..

[28] Malcangi G., Patano A., Morolla R., De Santis M., Piras F., Settanni V., Mancini A., Di Venere D., Inchingolo F., Inchingolo A.D. (2023). Analysis of Dental Enamel Remineralization: A Systematic Review of Technique Comparisons. Bioengineering.

[29] Dipalma G., Inchingolo A.D., Inchingolo A.M., Piras F., Carpentiere V., Garofoli G., Azzollini D., Campanelli M., Paduanelli G., Palermo A. (2023). Artificial Intelligence and Its Clinical Applications in Orthodontics: A Systematic Review. Diagnostics.

[30] Banning J.A. (1991). Chronic fatigue and shift work. Can. Nurse.

[31] Kalra Y. (2025). Chronotype and Nursing Shift Work. Am. J. Nurs..

[32] Tuominen O., Lundgren-Laine H., Teperi S., Salanterä S. (2020). Comparing the Two Techniques for Nursing Staff Rescheduling to Streamline Nurse Managers’ Daily Work in Finland. Comput. Inform. Nurs..

[33] Albert-Sabater J.A., Martínez J.M., Baste V., Moen B.E., Ronda-Perez E. (2016). Comparison of Menstrual Disorders in Hospital Nursing Staff According to Shift Work Pattern. J. Clin. Nurs..

[34] Inchingolo F., Santacroce L., Ballini A., Topi S., Dipalma G., Haxhirexha K., Bottalico L., Charitos I.A. (2020). Oral Cancer: A Historical Review. Int. J. Environ. Res. Public Health.

[35] Coffey L.C., Skipper J.K.J., Jung F.D. (1988). Nurses and Shift Work: Effects on Job Performance and Job-Related Stress. J. Adv. Nurs..

[36] Stimpfel A.W., Fatehi F., Kovner C. (2020). Nurses’ Sleep, Work Hours, and Patient Care Quality, and Safety. Sleep Health.

[37] Amritzer M.A., Muntlin Å., Berg L.M., Göransson K.E. (2021). Nursing Staff Ratio and Skill Mix in Swedish Emergency Departments: A National Cross-Sectional Benchmark Study. J. Nurs. Manag..

[38] Kalisch B.J., Lee H. (2009). Nursing Teamwork, Staff Characteristics, Work Schedules, and Staffing. Health Care Manag. Rev..

[39] Zverev Y.P., Misiri H.E. (2009). Perceived Effects of Rotating Shift Work on Nurses’ Sleep Quality and Duration. Malawi Med. J..

[40] Haghayegh S., Liu Y., Zhang Y., Strohmaier S., Papantoniou K., Markt S., Giovannucci E., Schernhammer E. (2023). Rotating Night Shift Work and Bladder Cancer Risk in Women: Results of Two Prospective Cohort Studies. Int. J. Environ. Res. Public Health.

[41] Inchingolo A.M., Patano A., Di Pede C., Inchingolo A.D., Palmieri G., de Ruvo E., Campanelli M., Buongiorno S., Carpentiere V., Piras F. (2023). Autologous Tooth Graft: Innovative Biomaterial for Bone Regeneration. Tooth Transformer^®^ and the Role of Microbiota in Regenerative Dentistry. A Systematic Review. J. Funct. Biomater..

[42] Malcangi G., Patano A., Ciocia A.M., Netti A., Viapiano F., Palumbo I., Trilli I., Guglielmo M., Inchingolo A.D., Dipalma G. (2023). Benefits of Natural Antioxidants on Oral Health. Antioxidants.

[43] Inchingolo F., Paracchini L., de Angelis F., Cielo A., Orefici A., Spitaleri D., Santacroce L., Gheno E., Palermo A. (2016). Biomechanical Behaviour of a Jawbone Loaded with a Prosthetic System Supported by Monophasic and Biphasic Implants. Oral Implantol..

[44] Minetti E., Dipalma G., Palermo A., Patano A., Inchingolo A.D., Inchingolo A.M., Inchingolo F. (2023). Biomolecular Mechanisms and Case Series Study of Socket Preservation with Tooth Grafts. J. Clin. Med..

[45] Bellocchio L., Inchingolo A.D., Inchingolo A.M., Lorusso F., Malcangi G., Santacroce L., Scarano A., Bordea I.R., Hazballa D., D’Oria M.T. (2021). Cannabinoids Drugs and Oral Health-From Recreational Side-Effects to Medicinal Purposes: A Systematic Review. Int. J. Mol. Sci..

[46] Parhizkar S., Holtzman D.M. (2025). The Night’s Watch: Exploring How Sleep Protects against Neurodegeneration. Neuron.

[47] Jørgensen J.T., Hansen J., Westendorp R.G.J., Nabe-Nielsen K., Stayner L.T., Simonsen M.K., Andersen Z.J. (2020). Shift Work and Incidence of Dementia: A Danish Nurse Cohort Study. Alzheimer’s Dement..

[48] Esmaily A., Jambarsang S., Mohammadian F., Mehrparvar A.H. (2022). Effect of Shift Work on Working Memory, Attention and Response Time in Nurses. Int. J. Occup. Saf. Ergon..

[49] Papantoniou K., Massa J., Devore E., Munger K.L., Chitnis T., Ascherio A., Schernhammer E.S. (2019). Rotating Night Shift Work and Risk of Multiple Sclerosis in the Nurses’ Health Studies. Occup. Environ. Med..

[50] Simunić A., Gregov L. (2012). Conflict between Work and Family Roles and Satisfaction among Nurses in Different Shift Systems in Croatia: A Questionnaire Survey. Arh. Hig. Rada Toksikol..

[51] Minelli A., Di Palma M., Rocchi M.B.L., Ponzio E., Barbadoro P., Bracci M., Pelusi G., Prospero E. (2021). Cortisol, Chronotype, and Coping Styles as Determinants of Tolerance of Nursing Staff to Rotating Shift Work. Chronobiol. Int..

[52] Feldman K., Rohan A.J. (2022). Data-Driven Nurse Staffing in the Neonatal Intensive Care Unit. MCN Am. J. Matern. Child. Nurs..

[53] Inchingolo F., Tatullo M., Abenavoli F.M., Marrelli M., Inchingolo A.D., Inchingolo A.M., Dipalma G. (2010). Comparison between traditional surgery, CO_2_ and Nd:Yag laser treatment for generalized gingival hyperplasia in Sturge-Weber syndrome: A retrospective study. J. Investig. Clin. Dent..

[54] Pisarski A., Bohle P., Callan V.J. (1998). Effects of Coping Strategies, Social Support and Work-Nonwork Conflict on Shift Worker’s Health. Scand. J. Work. Environ. Health.

[55] Min A., Kim Y.M., Yoon Y.S., Hong H.C., Kang M., Scott L.D. (2021). Effects of Work Environments and Occupational Fatigue on Care Left Undone in Rotating Shift Nurses. J. Nurs. Scholarsh..

[56] Sánchez Onrubia I.M., Resta Sánchez E.J., Cabañero Contreras T., Perona Moratalla A.B., Molina Alarcón M. (2025). Emergency Nursing Staff’s Well-Being, Burnout, and Sleep on 12-Hour Shifts. Enferm. Clin..

[57] Jørgensen J.T., Rozing M.P., Westendorp R.G.J., Hansen J., Stayner L.T., Simonsen M.K., Andersen Z.J. (2021). Shift Work and Incidence of Psychiatric Disorders: The Danish Nurse Cohort Study. J. Psychiatr. Res..

[58] Vitale E., Lupo R., Artioli G., Mea R., Lezzi P., Conte L., De Nunzio G. (2023). How Shift Work Influences Anxiety, Depression, Stress and Insomnia Conditions in Italian Nurses: An Exploratory Study. Acta Biomed..

[59] Dall’Ora C., Ejebu O.-Z., Ball J., Griffiths P. (2023). Shift Work Characteristics and Burnout among Nurses: Cross-Sectional Survey. Occup. Med..

[60] Camerino D., Conway P.M., Sartori S., Campanini P., Estryn-Béhar M., van der Heijden B.I.J.M., Costa G. (2008). Factors Affecting Work Ability in Day and Shift-Working Nurses. Chronobiol. Int..

[61] Kim M., Kim J.-H., Jung Y.W., Seong S.J., Kim S.-Y., Yoon H.-J., Lee S.-S., Kim H.-J., Ku B.-S., Cho H.-Y. (2022). Gynecologic Problems and Healthcare Behavior by Shift Patterns in Korean Nursing Staff. PLoS ONE.

[62] Stimpfel A.W., Aiken L.H. (2013). Hospital Staff Nurses’ Shift Length Associated with Safety and Quality of Care. J. Nurs. Care Qual..

[63] Berger A.M., Hobbs B.B. (2006). Impact of Shift Work on the Health and Safety of Nurses and Patients. Clin. J. Oncol. Nurs..

[64] Gonge H., Buus N. (2010). Individual and Workplace Factors That Influence Psychiatric Nursing Staff’s Participation in Clinical Supervision: A Survey Study and Prospective Longitudinal Registration. Issues Ment. Health Nurs..

[65] Saksvik-Lehouillier I., Bjorvatn B., Hetland H., Sandal G.M., Moen B.E., Magerøy N., Akerstedt T., Pallesen S. (2013). Individual, Situational and Lifestyle Factors Related to Shift Work Tolerance among Nurses Who Are New to and Experienced in Night Work. J. Adv. Nurs..

[66] Galanti T., Cortini M., Giudice G.F., Zappalà S., Toscano F. (2024). Safeguarding Nurses’ Mental Health: The Critical Role of Psychosocial Safety Climate in Mitigating Relational Stressors and Exhaustion. AIMS Public Health.

[67] Clark A., Moule P., Topping A., Serpell M. (2015). Rescheduling Nursing Shifts: Scoping the Challenge and Examining the Potential of Mathematical Model Based Tools. J. Nurs. Manag..

[68] Inchingolo F., Ballini A., Mura S., Farronato D., Cirulli N., Pettini F., Gheno E., Vermesan D., Pederzoli P., Resta G. (2015). Use of Platelet Rich Fibrin and Bio-OSS/SINT-Oss for Implant-Prosthetic Rehabilitation in Maxillary Atrophy with Sinus Pathology: A 48-Month Follow-Up. Eur. J. Inflamm..

[69] de Cordova P.B., Phibbs C.S., Stone P.W. (2013). Perceptions and Observations of Off-Shift Nursing. J. Nurs. Manag..

[70] Weng P.-W., Chang W.-P. (2025). Relationship between Shift Type and Sleep Quality in Rotating-Shift Nurses with Chronotype as a Moderator Variable. Int. Nurs. Rev..

[71] Salah R.A., Malak M.Z., Bani Salameh A.K. (2022). Relationship between Shift-Work and Life-Style Behaviors among Emergency Department Nurses in Jordan. Arch. Environ. Occup. Health.

[72] Silva-Costa A., Rotenberg L., Griep R.H., Fischer F.M. (2011). Relationship between Sleeping on the Night Shift and Recovery from Work among Nursing Workers—The Influence of Domestic Work. J. Adv. Nurs..

[73] Inchingolo F., Tatullo M., Pacifici A., Gargari M., Inchingolo A.D., Inchingolo A.M., Dipalma G., Marrelli M., Abenavoli F.M., Pacifici L. (2012). Use of Dermal-Fat Grafts in the Post-Oncological Reconstructive Surgery of Atrophies in the Zygomatic Region: Clinical Evaluations in the Patients Undergone to Previous Radiation Therapy. Head Face Med..

[74] Inchingolo F., Tatullo M., Abenavoli F.M., Marrelli M., Inchingolo A.D., Corelli R., Inchingolo A.M., Dipalma G. (2009). Upper Eyelid Reconstruction: A Short Report of an Eyelid Defect Following a Thermal Burn. Head Face Med..

[75] Inchingolo A.D., Patano A., Coloccia G., Ceci S., Inchingolo A.M., Marinelli G., Malcangi G., Di Pede C., Garibaldi M., Ciocia A.M. (2022). Treatment of Class III Malocclusion and Anterior Crossbite with Aligners: A Case Report. Medicina.

[76] Inchingolo A.M., Malcangi G., Costa S., Fatone M.C., Avantario P., Campanelli M., Piras F., Patano A., Ferrara I., Di Pede C. (2023). Tooth Complications after Orthodontic Miniscrews Insertion. Int. J. Environ. Res. Public Health.

[77] Niu S.-F., Chung M.-H., Chu H., Tsai J.-C., Lin C.-C., Liao Y.-M., Ou K.-L., O’Brien A.P., Chou K.-R. (2015). Differences in Cortisol Profiles and Circadian Adjustment Time between Nurses Working Night Shifts and Regular Day Shifts: A Prospective Longitudinal Study. Int. J. Nurs. Stud..

[78] Shiffer D., Minonzio M., Dipaola F., Bertola M., Zamuner A.R., Dalla Vecchia L.A., Solbiati M., Costantino G., Furlan R., Barbic F. (2018). Effects of Clockwise and Counterclockwise Job Shift Work Rotation on Sleep and Work-Life Balance on Hospital Nurses. Int. J. Environ. Res. Public Health.

[79] Dall’Ora C., Griffiths P., Emmanuel T., Rafferty A.M., Ewings S. (2020). The RN4CAST Consortium 12-hr Shifts in Nursing: Do They Remove Unproductive Time and Information Loss or Do They Reduce Education and Discussion Opportunities for Nurses? A Cross-sectional Study in 12 European Countries. J. Clin. Nurs..

[80] Inoue M., Takano M., Ueno C., Mori M., Morimatsu Y., Matsumoto Y., Kushino N., Ishitake T. (2020). Advantages of the Variable Shift System, and Effective Use of Break Time to Better Support the Work Engagement of Nurses on Extended Day Shifts. Kurume Med. J..

[81] Waage S., Pallesen S., Moen B.E., Vedaa Ø., Thun E., Vikanes Buchvold H., Blytt K.M., Harris A., Bjorvatn B. (2021). Changes in Work Schedule Affect the Prevalence of Shift Work Disorder among Norwegian Nurses—A Two Year Follow-up Study. Chronobiol. Int..

[82] Kubo T., Matsumoto S., Izawa S., Ikeda H., Nishimura Y., Kawakami S., Tamaki M., Masuda S. (2022). Shift-Work Schedule Intervention for Extending Restart Breaks after Consecutive Night Shifts: A Non-Randomized Controlled Cross-Over Study. Int. J. Environ. Res. Public Health.

[83] Jung H.-S., Lee B. (2015). Contributors to Shift Work Tolerance in South Korean Nurses Working Rotating Shift. Appl. Nurs. Res..

[84] Pahlevanzadeh M.J., Jolai F., Goodarzian F., Ghasemi P. (2021). A New Two-Stage Nurse Scheduling Approach Based on Occupational Justice Considering Assurance Attendance in Works Shifts by Using Z-Number Method: A Real Case Study. RAIRO-Oper. Res..

[85] Bülbül E., ÇeliK S., Özkan A., Akbaş G. (2023). Assessment of the Chronotypes of Nurses Working in Shifts and the Quality of Their Lives. Clin. Exp. Health Sci..

[86] Li J.-N., Chen X.-Q., Jiang X.-M., Zheng Q.-X., Pan Y.-Q., Zhu Y., Huang L., Liu R.-L. (2023). Exploring the Associations between Chronotype, Night Shift Work Schedule, Quality of Work Life, and Sleep Quality among Maternal and Child Health Nurses: A Multicentre Cross-Sectional Study. J. Nurs. Manag..

[87] De Bruijn L., Berentzen N.E., Vermeulen R.C.H., Vlaanderen J.J., Kromhout H., Van Leeuwen F.E., Schaapveld M. (2024). Chronotype in Relation to Shift Work: A Cohort Study among 37,731 Female Nurses. J. Sleep Res..

[88] Dehring T., Von Treuer K., Redley B. (2018). The Impact of Shift Work and Organisational Climate on Nurse Health: A Cross-Sectional Study. BMC Health Serv. Res..

[89] Abed Al Ahad M., Elbejjani M., Simon M., Ausserhofer D., Abu-Saad Huijer H., Dhaini S.R. (2022). Variability, Shift-specific Workloads and Rationed Care Predictors of Work Satisfaction among Registered Nurses Providing Acute Care: A Longitudinal Study. Nurs. Open.

[90] Lee J., Jeong I.S. (2021). Compliance with Recommendations on Work Schedule for Shift Nurses in South Korea. Saf. Health Work.

[91] Booker L.A., Mills J., Bish M., Spong J., Deacon-Crouch M., Skinner T.C. (2024). Nurse Rostering: Understanding the Current Shift Work Scheduling Processes, Benefits, Limitations, and Potential Fatigue Risks. BMC Nurs..

[92] Shin S.-H., Lee E.-H. (2024). Development and Validation of a Quality of Healthy Work Environment Instrument for Shift Nurses. BMC Nurs..

[93] Griepentrog J.E., Labiner H.E., Gunn S.R., Rosengart M.R. (2018). Bright Environmental Light Improves the Sleepiness of Nightshift ICU Nurses. Crit. Care.

[94] Bjorvatn B., Pallesen S., Waage S., Thun E., Blytt K.M. (2021). The Effects of Bright Light Treatment on Subjective and Objective Sleepiness during Three Consecutive Night Shifts among Hospital Nurses—A Counter-Balanced Placebo-Controlled Crossover Study. Scand. J. Work Environ. Health.

[95] Hoshi H., Iwasa H., Goto A., Yasumura S. (2022). Effects of Working Environments with Minimum Night Lighting on Night-Shift Nurses’ Fatigue and Sleep, and Patient Safety. BMJ Open Qual..

[96] Kim J.H., Song Y. (2020). The Effects of Indoor Ambient Temperature at Work on Physiological Adaptation in Night Shift Nurses. J. Nurs. Manag..

[97] Sadeghniiat-Haghighi K., Bahrami H., Aminian O., Meysami A., Khajeh-Mehrizi A. (2016). Melatonin Therapy in Shift Workers with Difficulty Falling Asleep: A Randomized, Double-Blind, Placebo-Controlled Crossover Field Study. Work.

[98] Gholipour Baradari A., Alipour A., Mahdavi A., Sharifi H., Nouraei S.M., Emami Zeydi A. (2018). The Effect of Zinc Supplementation on Sleep Quality of ICU Nurses: A Double Blinded Randomized Controlled Trial. Workplace Health Saf..

[99] Suyoto P.S., de Rijk M.G., de Vries J.H., Feskens E.J. (2024). The Effect of Meal Glycemic Index and Meal Frequency on Glycemic Control and Variability in Female Nurses Working Night Shifts: A Two-Arm Randomized Cross-Over Trial. J. Nutr..

[100] Leedo E., Beck A.M., Astrup A., Lassen A.D. (2017). The Effectiveness of Healthy Meals at Work on Reaction Time, Mood and Dietary Intake: A Randomised Cross-over Study in Daytime and Shift Workers at an University Hospital. Br. J. Nutr..

[101] Matsugaki R., Kuhara S., Saeki S., Jiang Y., Michishita R., Ohta M., Yamato H. (2017). Effectiveness of Workplace Exercise Supervised by a Physical Therapist among Nurses Conducting Shift Work: A Randomized Controlled Trial. J. Occup. Health.

[102] Baek Y., Han K., Kim J., Yoo H.Y. (2022). Smartphone-Based Home Workout Program for Shift-Work Nurses Working during the COVID-19 Pandemic. Nurs. Health Sci..

[103] Miyoshi Y. (2019). Restorative Yoga for Occupational Stress among Japanese Female Nurses Working Night Shift: Randomized Crossover Trial. J. Occup. Health.

[104] Baek G., Cha C. (2025). AI-Assisted Tailored Intervention for Nurse Burnout: A Three-Group Randomized Controlled Trial. Worldviews Evid. Based Nurs..

[105] Ell J., Brückner H.A., Johann A.F., Steinmetz L., Güth L.J., Feige B., Järnefelt H., Vallières A., Frase L., Domschke K. (2024). Digital Cognitive Behavioural Therapy for Insomnia Reduces Insomnia in Nurses Suffering from Shift Work Disorder: A Randomised-Controlled Pilot Trial. J. Sleep. Res..

[106] Liu W., Wang Q., Zheng D., Mei J., Lu J., Chen G., Wang W., Ding F. (2024). The Effects of a Complex Interactive Multimodal Intervention on Personalized Stress Management Among Health Care Workers in China: Nonrandomized Controlled Study. J. Med. Internet Res..

[107] Lu C., Sun Y., Wang C., Chen T., Tang Y. (2025). The Effects of Confiding on Shift Work Nurses’ Emotion Regulation and Self-Perceived Well-Being: An Online Randomized Controlled Trial. Behav. Sci..

[108] Watanabe K., Sugimura N., Shishido I., Konya I., Yamaguchi S., Yano R. (2022). Effects of 90 Min Napping on Fatigue and Associated Environmental Factors among Nurses Working Long Night Shifts: A Longitudinal Observational Study. Int. J. Environ. Res. Public Health.

[109] Watanabe K., Shishido I., Ito Y.M., Yano R. (2025). Quantity and quality of napping to mitigate fatigue and sleepiness among nurses working long night shifts: A prospective observational study. J. Physiol. Anthropol..

[110] Booker L.A., Sletten T.L., Barnes M., Alvaro P., Collins A., Chai-Coetzer C.L., McMahon M., Lockley S.W., Rajaratnam S.M.W., Howard M.E. (2022). The Effectiveness of an Individualized Sleep and Shift Work Education and Coaching Program to Manage Shift Work Disorder in Nurses: A Randomized Controlled Trial. J. Clin. Sleep Med..

[111] Albakri U., Smeets N., Kant I., Meertens R. (2024). Strategies That Nurses Working Irregular Night Shifts Use to Improve Sleep Quality: A Qualitative Study among Good and Poor Sleepers. J. Adv. Nurs..

[112] Oriyama S., Miyakoshi Y., Rahman M.M. (2019). The Effects of a 120-Minute Nap on Sleepiness, Fatigue, and Performance during 16-Hour Night Shifts: A Pilot Study. J. Occup. Health.

[113] Comparative Effects of Music Therapy and Aromatherapy on Stress, Quality of Life, and Happiness Among Shift Nurses in Korea: A Randomized Controlled Trial. https://jkbns.org/journal/view.php?number=657.

[114] Wang X., Feng T., Liu S., Ruan J. (2024). Application of Music Therapy in Improving the Sleep Quality and Mental Health of Nurses with Circadian Rhythm Sleep Disorders Caused by Work Shifts. Noise Health.

[115] Zamanifar S., Bagheri-Saveh M.I., Nezakati A., Mohammadi R., Seidi J. (2020). The Effect of Music Therapy and Aromatherapy with Chamomile-Lavender Essential Oil on the Anxiety of Clinical Nurses: A Randomized and Double-Blind Clinical Trial. J. Med. Life.

[116] Molzof H.E., Wirth M.D., Burch J.B., Shivappa N., Hebert J.R., Johnson R.L., Gamble K.L. (2017). The Impact of Meal Timing on Cardiometabolic Syndrome Indicators in Shift Workers. Chronobiol. Int..

[117] Nasiri A., Boroomand M.M. (2021). The Effect of Rosemary Essential Oil Inhalation on Sleepiness and Alertness of Shift-Working Nurses: A Randomized, Controlled Field Trial. Complement. Ther. Clin. Pract..

